# Climate change and commercial fishing practices codetermine survival of a long‐lived seabird

**DOI:** 10.1111/gcb.16482

**Published:** 2022-10-22

**Authors:** Daniel Gibson, Thomas V. Riecke, Daniel H. Catlin, Kelsi L. Hunt, Chelsea E. Weithman, David N. Koons, Sarah M. Karpanty, James D. Fraser

**Affiliations:** ^1^ Graduate Degree Program in Ecology, Department of Fish, Wildlife, and Conservation Biology Colorado State University Fort Collins Colorado USA; ^2^ Department of Fish and Wildlife Conservation Virginia Tech Blacksburg Virginia USA; ^3^ Swiss Ornithological Institute Sempach Switzerland

**Keywords:** capture‐recapture, ecological networks, hierarchical models, *Thalasseus maximus*, top‐down and bottom‐up population regulation

## Abstract

Understanding the environmental mechanisms that govern population change is a fundamental objective in ecology. Although the determination of how top‐down and bottom‐up drivers affect demography is important, it is often equally critical to understand the extent to which, environmental conditions that underpin these drivers fluctuate across time. For example, associations between climate and both food availability and predation risk may suggest the presence of trophic interactions that may influence inferences made from patterns in ecological data. Analytical tools have been developed to account for these correlations, while providing opportunities to ask novel questions regarding how populations change across space and time. Here, we combine two modeling disciplines—path analysis and mark‐recapture‐recovery models—to explore whether shifts in sea‐surface temperatures (SSTs) influenced top‐down (entanglement in fishing equipment) or bottom‐up (forage fish production) population constraints over 60 years, and the extent to which these covarying processes shaped the survival of a long‐lived seabird, the Royal tern. We found that hemispheric trends in SST were associated with variation in the amount of fish harvested along the Atlantic coast of North America and in the Caribbean, whereas reductions in forage fish production were mostly driven by shifts in the amount of fish harvested by commercial fisheries throughout the North Atlantic the year prior. Although the indirect (i.e., stock depletion) and direct (i.e., entanglement) impacts of commercial fishing on Royal tern mortality has declined over the last 60 years, increased SSTs during this time period has resulted in a comparable increase in mortality risk, which disproportionately impacted the survival of the youngest age‐classes of Royal terns. Given climate projections for the North Atlantic, our results indicate that threats to Royal tern population persistence in the Mid‐Atlantic will most likely be driven by failures to recruit juveniles into the breeding population.

## Graphical abstract

Commercial fishing intensity (a) was directly (via entanglement risk associated with long‐line fishing) and indirectly (via reductions in Atlantic menhaden production associated with commercial trawling) link to increased risk of Royal tern mortality. Although (b) sub‐adult (ages 3–4) and adult (age >5) survival has increased over time partially as a function of reductions in pressures related to commercial fishing, survival of the youngest age‐class of Royal terns was primarily associated with increased sea‐surface temperatures in the North Atlantic.

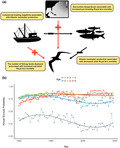

## 1 INTRODUCTION

An understanding of the driving forces of population trends and individual fitness is a key component of conservation ecology (Sibley & Hone, 2002). Demographic data are often the foundation of our understanding of these forces, and the most effective applied management actions target problematic points in the life cycle (Nichols et al., 2007). Detailed assessments of population drivers are urgently needed across taxa, as climate change is projected to have important impacts on populations and communities globally (Peterson et al., 2021; Thomas et al., 2004; Urban, 2015). Drivers of population dynamics often are synergistic (Brook et al., 2008), where multiple mechanisms may have compounding effects on population declines. Critically, ecological drivers often co‐vary, and ecological variables may affect each other as much or more than demographic response variables of interest. These processes lead to both direct and indirect effects of system change. Properly linking relationships among top‐down and bottom‐up system drivers, as well as other ecological covariates (e.g., population sex or age ratios), will allow for the most effective biological inference and decisions at population and community levels. Finally, explicitly linking changing system components to the demographic components that drive population change (Koons et al., 2016; Manlik et al., 2018) will further increase the effectiveness of conservation actions.

The mechanisms limiting population growth can be coarsely grouped as either a resource limitation (i.e., bottom‐up) or through species interactions (i.e., top‐down or competition; Hunter & Price, 1992). Although the ecological literature is populated with theoretical and empirical support for either top‐down (Banse, 2007; Hairston et al., 1960) or bottom‐up (Villar et al., 2014; White, 1978) effects being the predominant constraint on population dynamics, it is likely that the forces regulating populations are themselves variable over time. This results in populations potentially being co‐limited by competing, and potentially asynchronous, pressures (Meserve et al., 2003; Power, 1992). However, from a conservation perspective, management actions may often only prioritize either top‐down processes (e.g., harvest regulations, predator removal), or bottom‐up processes (often indirectly through habitat management), which limits the scope of potential inference, system inputs, and outcomes (Manlik et al., 2018).

Globally, seabirds are one of the most threatened avian taxa, with nearly half of all species exhibiting population declines (Croxall et al., 2012). Mortality associated with fishery bycatch, climate change, invasive species, and overfishing are the four most ubiquitous threats faced by seabirds (Dias et al., 2019). Specific to seabirds in the North Atlantic, many of the prey species historically available for seabirds (e.g., Atlantic menhaden [*Brevoortia tyrannus*], Atlantic herring [*Clupea harengus*], Atlantic silversides [*Menidia menidia*], capelin [*Mallotus villosus*]; Aygen & Emslie, 2006) have exhibited range contractions or declines in abundance possibly driven by climate change (Buchheister et al., 2016; Buren et al., 2014; Essington et al., 2015; Hughes et al., 2015; Roberts et al., 2019). In addition to driving reductions in fish abundance, shifts in water temperature may influence both the geographic (Roberts et al., 2020; Rose, 2005) and depth (Dulvy et al., 2008) distributions fish occupy, as well as the growth rates (Heather et al., 2018; Rountrey et al., 2014), and movement speed (Annis et al., 2011) of fish. Taken together, these changes may influence foraging effort and efficiency of seabirds and other predators (Grémillet & Boulinier, 2009) as well as seabird population dynamics (Kowalczyk et al., 2015).

**FIGURE 1:**
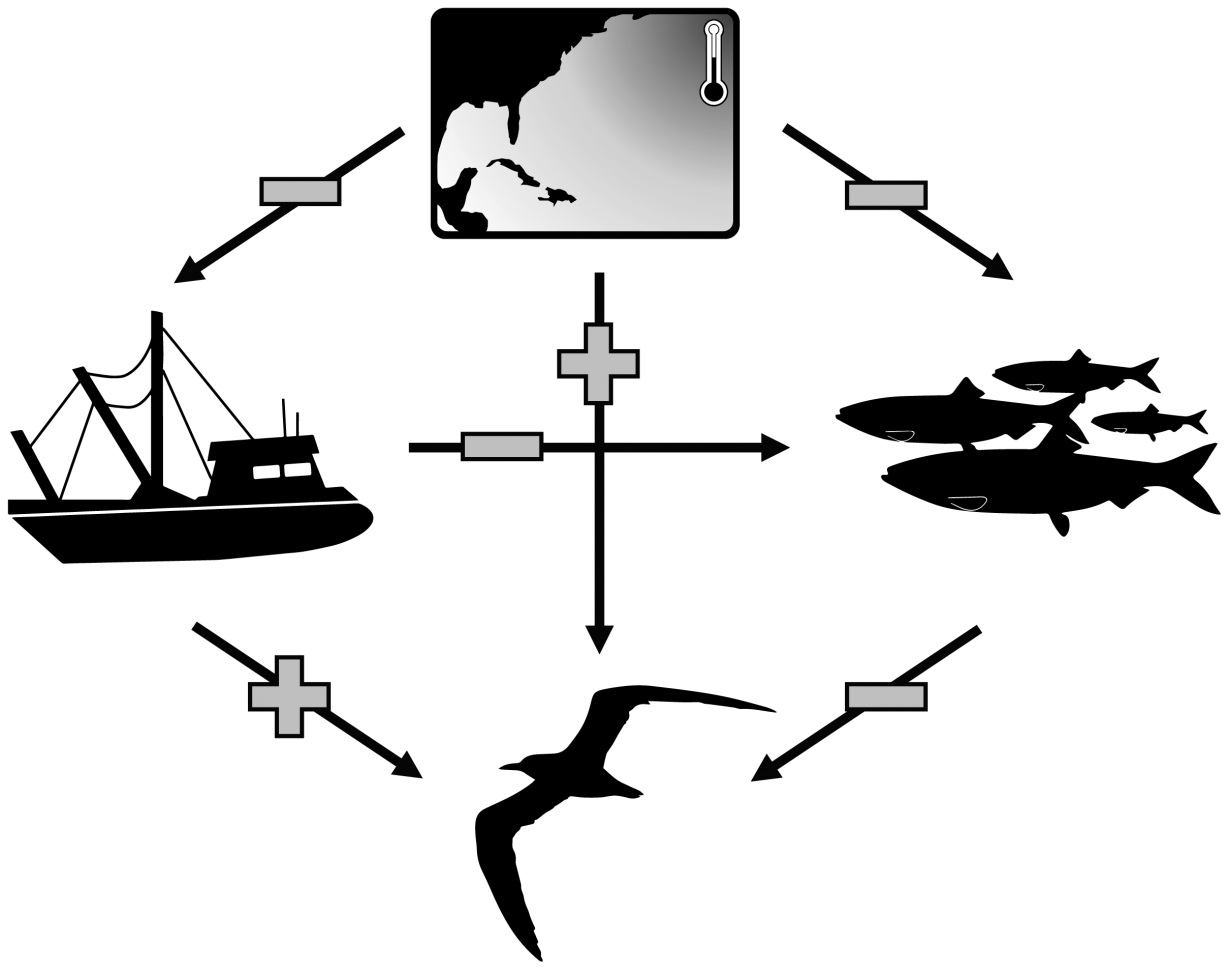
Simplified path diagram describing the hypothesized directionality (plus: Positive, bar: Negative association) regarding how environmental variables (i.e., sea‐surface temperatures in the North Atlantic, fishery pressure, and fish production) influenced one another, as well as the indirect and direct pathways in which these sources of environmental variability influenced Royal tern mortality.

From a top‐down perspective, seabirds are attracted to fishing boats that target their prey (Wickliffe & Jodice, 2010); therefore, seabirds are susceptible to injury and mortality related to both active and remnant (e.g., discarded fishing line) equipment associated with commercial and recreational fishing (Devney et al., 2009). Thus, fishing acts as an introduced top‐down pressure, which may be either correlated with, or directly affect the health of regional fisheries (Mullon et al., 2005). Likewise, climate‐related shifts in fish behavior, distribution, or population dynamics may be exacerbated by commercial fishing through a variety of mechanisms including overfishing (Essington et al., 2015) or altering the phenotypic composition of a population (Morrongiello et al., 2019). Because climate change and commercial fishing both have the potential to independently alter predator–prey dynamics between seabirds and fish, a detailed understanding of each of these processes is important for seabird conservation (Barbraud et al., 2012). Put simply, seabird populations may experience bottom‐up pressures associated with climate change and prey availability, and top‐down pressures associated with commercial fishing practices, which may itself be affected by changes in fish distribution and abundance. Therefore, understanding how environmental conditions are inherently connected is required for reliable inference into the individual importance of multiple sources of environmental variability on seabird population dynamics (Figure 1).

Royal terns (*Thalasseus maximus*; hereafter ROYT) are a long‐lived seabird that primarily nest in large breeding colonies on islands throughout the Atlantic, Gulf of Mexico, and Pacific coasts of North America, with a non‐breeding range that extends into the Caribbean and coastal South America (Buckley et al., 2021). Although not considered to be a species of conservation concern, regional ROYT populations throughout the United States have declined in abundance since the 1970s (Emslie et al., 2009; Foster et al., 2009; Jodice et al., 2007). Similar to many other seabirds, habitat degradation, reductions in food availability, and incidental take associated with fishing are considered likely drivers of regional population declines (Buckley et al., 2021). Further, given the high apparent survivorship (*φ*) of adults (*φ* = 0.95 [Collins & Doherty Jr, 2006], 0.68 [Liechty et al., 2017]) and fixed clutch size (one egg, no re‐nesting, Buckley et al., 2021), regional population declines would most likely have been driven by either shifts in colony attendance (e.g., breeding propensity or site fidelity) or post‐initiation reproductive failure (e.g., nest or juvenile survival). Given that our current knowledge regarding ROYT survival is limited to a few short‐term studies (Collins & Doherty Jr, 2006; Liechty et al., 2017) that lacked the power to determine ecological patterns, an assessment into the drivers of population dynamics for this species was warranted.

Here, we use 60 years of live‐resights and dead‐recoveries of nearly 650,000 ROYT banded in the mid‐Atlantic region of the United States, in conjunction with time series data describing shifts in environmental conditions to construct an ecological network to codetermine whether (1) annual variation in fish population trajectories, fishery harvest pressure, and climate conditions were correlated with one another and (2) whether these sources of environmental variability directly or indirectly influenced age‐specific survival of ROYT. Additionally, this parameterization may serve as a guiding framework for evaluating the impacts of conservation actions given the behavior of complex ecological systems in the face of climate change (Grace et al., 2015). In addition to the proposed advancements in demographic inference, we jointly aim to improve the understanding of ROYT population dynamics as a whole given the relative absence of a detailed description of ROYT demographic rates in the literature.

## 2 MATERIALS AND METHODS

### 2.1 Study system and data collection

#### 2.1.1 Mark‐recapture data

**FIGURE 2:**
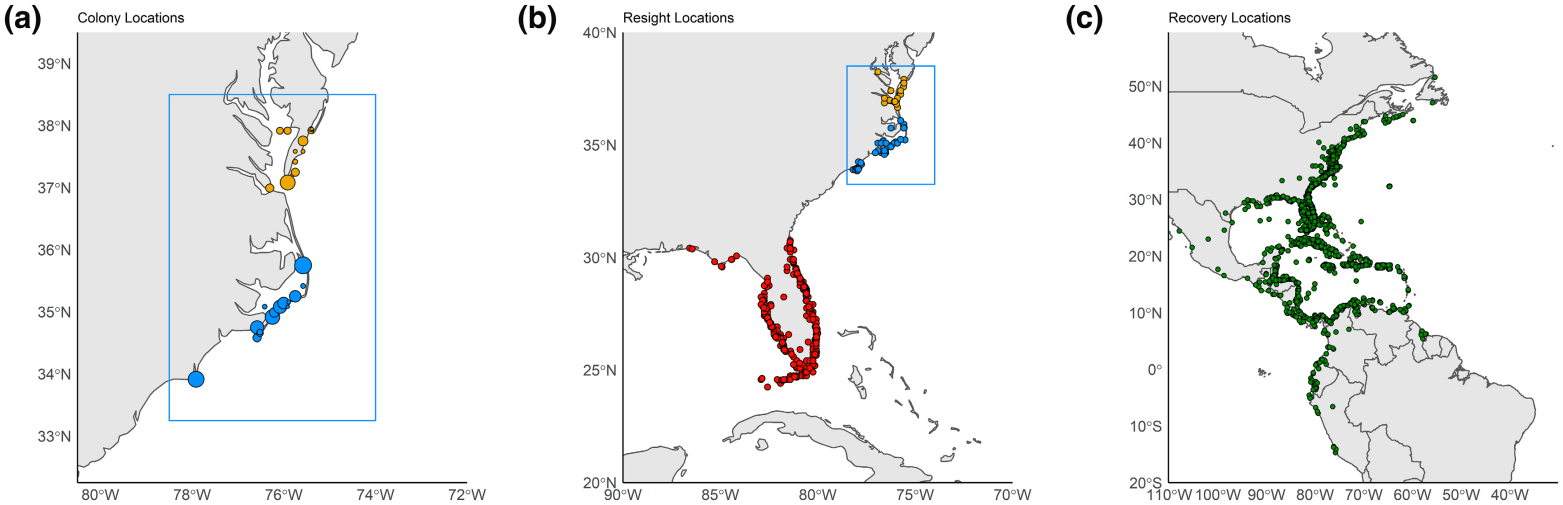
Map of the locations of all (a) colony locations in North Carolina (blue) or Virginia (yellow) where Royal terns were banded as flightless chicks, (b) reencounter locations of individuals during the breeding season (June–August) or in Florida (red) during the non‐breeding season (October–April), and (c) recoveries locations of dead individuals reported to the Bird Banding Lab throughout the western hemisphere from 1961 to 2021.

ROYT have been extensively banded on the breeding grounds in the mid‐Atlantic region of North America as part of multiple research and monitoring activities (e.g., Buckley & Buckley, 1977; Emslie et al., 2009; Erwin, 1978; Figure 2). From these researchers' efforts, we collated banding, live‐resight, and dead‐recovery data for 646,150 ROYT banded as flightless chicks (1–4 weeks of age) from 1961 to 2020 across approximately two dozen nesting colonies located in coastal North Carolina and Virginia of the United States. Chicks were initially marked through organized banding drives that occurred from late June through mid‐July, in which researchers walked through the nesting colony and herded the creche of chicks into a previously constructed corral. Following capture, chicks large enough to retain a band, were fit with a standard issue U.S. Fish and Wildlife Service band (hereafter, service band) and released. During 10 years of the study (1968, 1970, 1976–1978, 2000–2003, 2007) a subset (*N* = 7432) of chicks were fit with either a secondary plastic or metal band designed to improve detectability (hereafter, early ancillary bands). Likewise, during the final 4 years of banding efforts in Virginia (2018–2021), a subset (*N* = 7401) of chicks were fit with a larger and, in theory, even more detectable three‐digit alpha‐numeric plastic band (hereafter, plastic field readable band [PFR]).

Reencounters of marked individuals were generated by both the public and structured research efforts on ROYT through resighting (or recaptures) of living individuals, or the recovery of dead individuals that were reported to the Bird Banding Lab (Patuxent, MD; hereafter, BBL). Reencounters of previously marked individuals were broken into discrete classifications, which included being (1) observed alive in North Carolina during the breeding season (June–July), (2) observed alive in Virginia during the breeding season (June–July), (3) observed alive in Florida during the non‐breeding season (August–May), or (4) recovered dead throughout the global range of the species during the entire year. We limited the geographic extent of non‐breeding resights to those reported in Florida to reduce overdispersion in these data and to ensure that non‐breeding resights occurred after migration from the breeding grounds. Following the exclusion of reencounter records where an individual's status (alive or dead) or the timing/location of an observation could not be discerned, we retained resight/recovery records for 14,504 individuals (~2% of birds released) for which a live reencounter (*n* = 7191 [NC = 838; VA = 1256; FL = 5097]) or dead recovery (*n* = 7814) was reported to the BBL. Although the cause of death for most reported recoveries was unknown, the most frequent cause of ‘known’ mortality of ROYT reported to the BBL was related to entanglement with fishing gear. Likewise, previous research has estimated that entanglement accounts for ~20% of reported recoveries, and indicated pre‐breeding and sub‐adult ROYT were at an increased risk of entanglement (Buckley & Buckley, 1974; Sanders & Ray, 2015).

#### 2.1.2 Climate data

We used the average (October–March) sea‐surface temperature (SST) in the North Atlantic basin (latitude: 0°–50°N; longitude: −90°–0° W) for each year from 1950 to 2020 to describe annual variation in climate conditions during the ROYT non‐breeding season (Huang et al., 2017). Within most of our study system, increased SSTs would also be associated with wetter conditions and would allow warmer, more saline waters to travel farther northward (Roberts et al., 2019). Whereas, lower SSTs would be associated with colder, dryer conditions, and less overturning of warmer, more saline waters northward.

#### 2.1.3 Fisheries data

We used data describing annual variation in the number of hooks deployed and total landings by commercial and artisanal fisheries throughout the Atlantic Ocean as indices of harvest pressure (see *Commercial and artisanal harvest*). As the amount of fish harvested in a year may also be related to the overall health of regional fisheries, we also included the estimated relative abundances of 1‐year old Atlantic menhaden (*B. tyrannus*), Atlantic red drum (*Sciaenops ocellatus*), and Atlantic herring (*C. harengus*) to disentangle the effects of shifts in relative prey abundance versus the risk of entanglement (see *Population trends of potential prey*).

##### Commercial and artisanal harvest

Information regarding spatiotemporal variation in the annual catch amounts of fish in the Atlantic Ocean were obtained from Sea Around Us (Zeller et al., 2016; http://www.seaaroundus.org), which combined harvest data reported to the Food and Agriculture Organization of the United Nations with ancillary data and predictions of unreported bycatch and discarded fish. We used the annual tonnage of fish and ocean invertebrates landed by (1) commercial long‐line fishing, pole‐line, hand‐line, and gill‐netting and (2) commercial trawl‐based fishing along the Atlantic coast of the United States from 1950 to 2015 as indices of temporal variation in entanglement risk along the Atlantic Coast for two distinct fisheries. Given that commercial fishing was not prominent in the Caribbean, we used estimated (3) annual tonnage of fish and ocean invertebrates landed by artisanal long‐line fishing, pole‐line, hand‐line, and all forms of net‐based fishing in the waters surrounding The Bahamas, Cuba, Dominican Republic, and Jamaica, which were the regions within the Caribbean associated with the greatest amount of take from 1950 to 2015. We separated the amount of harvest associated with trawl‐based fishing from long‐line and net‐based fishing, as trawling was orders of magnitude greater in tonnage harvested, relative to commercial and artisanal long‐line fishing, as well as differed in the taxa targeted and gear used, and therefore the potential risk to ROYT. Lastly, as the gear associated with long‐line fishing has been previously associated with ROYT entanglement and mortality (Buckley & Buckley, 1974; Sanders & Ray, 2015), we included (4) estimates of the number of hooks deployed each year in the North Atlantic and Gulf of Mexico collected in adherence to the fishery logbook system (Garrison & Stokes, 2021) to provide insights into the extent that fishery harvest and gear deployment were correlated and the direct impacts of fishing gear on ROYT mortality. However, estimates of the number of hooks deployed in the north Atlantic and Gulf of Mexico were only available from 1993 to 2019.

##### Population trends of potential prey

Atlantic menhaden, Atlantic red drum, and Atlantic herring were chosen as representatives, not an exhaustive list, of potential prey items of ROYT as they were (1) demonstrated to be a consistent part of their breeding‐season diet (Aygen & Emslie, 2006; Erwin, 1977; Liechty et al., 2016; Wambach & Emslie, 2003), (2) relatively abundant across a substantial part of the breeding and non‐breeding ranges of ROYT, and (3) associated with stock assessments across multiple decades that were generated in similar manner. For this analysis, we used estimates of the abundance of 1‐year‐old fish for species from 1965 to 2014 based on a combination of fishery‐dependent sampling and fishery‐independent surveys (description and data can be found in Deroba, 2015; SEDAR, 2015, 2020) as indices of temporal variation in prey availability. Although adult ROYT can forage for larger size classes, other fish species, and other ocean invertebrates, this age‐class for these three fish species was selected as it included the dietary size breadth for feeding chicks (~30–130 mm; Aygen & Emslie, 2006) as well as larger fish potentially required for individual maintenance.

### 2.2 Analytical methods

#### 2.2.1 Environmental sub‐model

The environmental model estimated the extent to which environmental variables (i.e., climate, fishery harvest/pressure, and fish production) covaried with or influenced other environmental variables across time. We modeled associations between environmental variables in a regression‐based framework to assess our a priori beliefs about the directionality of each relationship. We hypothesized that (1) SSTs negatively influenced both the amount of fish harvested and fishery production (Buchheister et al., 2016; Hughes et al., 2015), (2) fish production in the Atlantic was negatively impacted by a lagged effect of the amount of landings from commercial fishing along the eastern continental shelf of the United States (Buchheister et al., 2016), and (3) the number of hooks deployed by the long‐line fishing industry was positively correlated with amount of landings from long‐line fishing, and we developed a model that tested these assumptions (Equations (1), (2), (3), (4)).

We modeled annual variation in log‐scaled artisanal harvest of fish in the Caribbean (CR) or commercial harvest along the eastern United States by line or nets (LI) or trawling (TR) as a function of (1) current SST conditions, (2) a quadratic time trend ($\beta_{T}$ + $\beta_{T^{2}}$), and (3) the observed deviation in harvest pressure (${\text{HARV}}_{j,t-1}$) from the estimated value ($\mu_{j,t-1}$) the year prior (Equation 1) represented by a first‐order autoregressive (AR1) parameter ($\varphi_{j}$) to account for serial autocorrelation in landings. Lastly, and specific to the Atlantic long‐line fishing model, we included a correlation between the estimated pelagic longline fishing effort (annual number of hooks deployed; $\beta_{\mathrm{LL}}\rho {\mathrm{hks}}_{t}$) and estimated landings of fish from long‐line fishing along the Atlantic Coast of North America and Gulf of Mexico to explore whether trends in landings for this fishery was primarily related to fish abundance or fishery effort.

(1) $$\begin{array}{c} \mu_{j,t}=\beta_{0,j}+\beta_{T,j}t+\beta_{T^{2},j}t^{2}+\beta_{S,j}{\mathrm{SST}}_{t}+\varphi_{j}\left(\mu_{j,t-1}-{\text{HARV}}_{j,t-1}\right)\\ +\beta_{\mathrm{LL}}\rho {\mathrm{hks}}_{t}+\varepsilon_{\text{harv}j,t}, \end{array}$$

$$\log\left({\text{HARV}}_{j,t}\right)∼\text{Normal}\left(\mu_{j,t,}\sigma_{j}\right)$$

The correlation ($\rho {\mathrm{hks}}_{t}$) between the number of hooks deployed from commercial long‐line fishing and landings via long‐line fishing was modeled as a latent process (Normal distribution with a mean of 0 and *σ* of 1) jointly informed by both time series. As the data describing fluctuations in hook deployment were not available prior to 1993, we used the estimated correlation between landings and hook deployment and a first‐order autoregressive model to predict hook deployment from 1961 to 1992 (Equation 2).

(2) $$\rho {\mathrm{hks}}_{t}∼\text{Normal}\left(0,\sigma =1\right)$$

$${\text{Hooks}}_{t}∼\text{Normal}\left(\mu_{\mathrm{hk},t},\sigma_{\mathrm{hk}}\right)$$

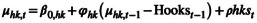




Likewise, we modeled annual variation in the log‐scaled abundance of 1‐year‐old Atlantic menhaden (MN), red drum (DM), and herring (HR) as a function of (1) current SST, (2) lagged effects for the tonnage of landings associated with trawling ($\beta_{\mathrm{TR}}$) or long‐line ($\beta_{\mathrm{LI}}$) fishing along the eastern United States the previous year, (3) a quadratic time trend ($\beta_{T}$ + $\beta_{T^{2}}$), and (4) an autoregressive effect of the observed fish production the year prior (Equation 3),

(3) $$\begin{array}{c} \mu_{j,t}=\beta_{0,j}+\beta_{T,j}t+\beta_{T^{2},j}t^{2}+\beta_{S,j}{\mathrm{SST}}_{t}+\beta_{\mathrm{TR},j}{\mathrm{TR}}_{t-1}\\ +\beta_{\mathrm{LI},j}{\mathrm{LI}}_{t-1}+\varphi_{j}\left(\mu_{j,t-1}-{\text{FISH}}_{j,t-1}\right)+\varepsilon_{\text{fish}j,t} \end{array}$$

$$\log\left({\text{FISH}}_{j,t}\right)∼\text{Normal}\left(\mu_{j,t,}\sigma_{j}\right).$$

We did not model the impact of artisanal fisheries throughout the Caribbean on fish production, as the geographic range of these fish species did not sufficiently extend into the Caribbean. We modeled the extent to which environmental variables of the same type (i.e., variables associated with either fish production ($\varepsilon_{\text{fish}}$) or fishery pressure($\varepsilon_{\text{harv}}$)) covaried with each other variable of the same type using methods described in Barnard et al. (2000) where the variance–covariance matrix that informed the residual error term for each group of variables was composed of separate priors for standard deviations and associated correlation matrices ($\Sigma_{\text{fish}}$, $\Sigma_{\text{harv}}$).

(4) $$\varepsilon_{\text{fish}j,t}∼\text{Multivariate Normal}\left(0,\Sigma_{\text{fish}1:3,1:3}\right),$$

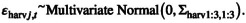




The time series data for both the fisheries harvest and fish production were incomplete relative to the temporal extent of the ROYT demographic data. As such, we generated predictions for each explanatory variable during years in which data were not available (landings: 2016–2020; production: 1961–1964; 2016–2020; hooks: 1961–1992) from the linear models associated with each variable, which included the estimated covariances among variables. Lastly, a quadratic time trend was selected as preliminary data exploration suggested it reduced violations of the normality assumption relative to models with a linear or no time trend.

#### 2.2.2 Demographic sub‐model

We used the marginalized likelihood (Turek et al., 2016; Yackulic et al., 2020) parameterization of a multistate Barker model (Barker, 1997; Riecke et al., 2021), which was modified to allow for (1) age‐specific (1–25+ years) estimates of true survival (S); (2) two spatial‐scales of fidelity to the breeding population (i.e., fidelity to the mid‐Atlantic metapopulation [F]; and fidelity to state‐specific [NC vs. VA] breeding population [*ψ*]); and (3) proportional (see Equation 9) breeding propensity (α), which accounted for temporal variation in breeding detection/recapture (*p*), non‐breeding detection (*R*, *R'*), and dead recovery (*r*) probabilities.

The process matrix ($\Psi_{i,j}$; Supplemental Material) for the multistate Barker model included three living states (1: alive, in the NC breeding population; 2: alive, in the VA breeding population; and 3: alive, but permanently emigrated from the mid‐Atlantic [NC and VA] metapopulation during or prior the most recent timestep) and three dead states (4: recovered and reported as dead during most recent timestep; 5: died during the most recent timestep, but was not resighted; and 6: died during a previous timestep). Here, transitions among the living states were a function of survival (*S*) and the two movement parameters (*F* and *ψ*), where *F* represented the proportion of each surviving breeding population that did not permanently leave the mid‐Atlantic metapopulation between two breeding seasons and *ψ* represented the proportion of each surviving breeding population that remained in the state (NC or VA) that they were associated during the previous breeding season. Transitions from the living states to the dead states were a function of the probability of mortality during a specific timestep (1 − *S*) and the probabilities of being recovered during that timestep (*r*) or being encountered alive during, but prior to death, the non‐breeding season an individual ultimately died (*R'*). Transitions among dead states were unidirectional (i.e., from each dead state to the “previous dead” or terminal dead state) and fixed to one.

Age‐specific (*a*) survival probabilities ($S_{a,t}$) were parameterized as the log–log transformation of year‐specific, *t*, mortality risk functions (Equation 5).

(5) $$S_{a,t}=\exp\left(-\exp\left(\varepsilon_{A,t}\right)\right)$$

For the survival model, individuals were assigned into three age‐classes that were defined as (1) first and second year (hereafter, pre‐breeding), (2) third and fourth year (hereafter, sub‐adult), and (3) fifth year and older individuals (hereafter, adults). ROYT exhibit delayed recruitment into the breeding population, in which no individuals have been observed breeding before their third year, and breeding propensity is believed to be reduced, relative to mature adults, until at least an individual's fifth year (J. Weske, *personal communication*). Thus, these age‐classes broadly separated individuals into groups as a function of their reproductive status, which may influence their relative susceptibility to different mortality hazards due to life history trade‐offs, variation in seasonal ranges, or learned experience. Temporal variation ($\varepsilon_{A,t}$) in age‐specific mortality risk was modeled as the outcome of a multivariate normal distribution centered on the linear mortality risk model (${\bar{S}}_{A,t}$) for each three age‐class, *A*, and corresponding variance–covariance matrix ($\Sigma_{A}$). We allowed the intercepts ($\beta_{0A}$) [prior: conjugate log(exponential(1))] and beta coefficients (β) for each covariate to be estimated independently for each age‐class and included previously described variation in SSTs, fishery pressure (Harv) and fishery production (Fish) as covariates. We parameterized β with a lasso regularizing prior to reduce overfitting and improve convergence

$$\varepsilon_{A,t}∼\text{Normal}\left({\bar{S}}_{A,t},\Sigma_{1:3,1:3}\right)$$

βx,A∼double exponential0,1υA

$$\upsilon_{A}∼\text{exponential}\left(.1\right)$$

(6)

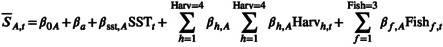




Although the model accounted for temporal variation in mortality risks among three age classes, we also included a series of intercept adjustment terms ($\beta_{a}$) that allowed for mortality rates to differ as an individual aged. Although the oldest individual reencountered in the dataset was 31 years old, 99% of all reencounters/recoveries were from individuals younger than 24 years of age; thus, we constrained all individuals older than 25 to jointly inform oldest age (i.e., 25) due to data limitation concerns.

Annual fluctuations in recovery probabilities (*r*) were modeled as a function of environmental and residual variation to account for potential overdispersion due to the sparseness of these data. We included the fishery harvest pressure and fish production variables mentioned above to account for whether variation in harvest pressure or fish conditions influenced where and how ROYT were dying, which may influence the probability of being recovered/reported by a human. Additional overdispersion in recovery probabilities were accounted for through a hierarchical random effects model centered on the linear recovery model (${\bar{r}}_{t}$) with an error term ($\sigma_{r}$) [prior: *half‐Cauchy* (*τ* = 0.16)]. We included an intercept adjustment term ($\beta_{rS}{\mathrm{NC}}_{i}$) that allowed recovery rates to differ between individuals banded in NC relative to those banded in VA. Lastly, we considered an intercept‐adjustment term for the band type assigned to an individual ($\beta_{rb}$) [i.e., (1) metal‐only, (2) early ancillary markers, or (3) plastic field‐readable markers] to account for variation in either the probability of recovery or reporting a dead individual associated with the visibility of its band.

$$\text{logit}\left(r_{b,t}\right)∼\text{Normal}\left({\bar{r}}_{b,t},\sigma_{r}\right)$$

(7)

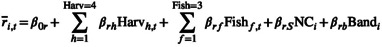




We constrained fidelity to the mid‐Atlantic metapopulation (*F*) and within the mid‐Atlantic metapopulation (*ψ*) to be constant across space and time as preliminary models indicated both traits were high and models that considered temporal variation suffered from boundary estimation issues. However, we did not allow individuals to permanently emigrate from the mid‐Atlantic breeding population, or move from the state of their birth until they reached the minimum breeding age for the species (i.e., 4 years old).

Annual variation in $R_{t}$ was modeled as a random effect, centered on the logit‐scaled mean non‐breeding resight rate ($\bar{R}$ [prior: logistic(0,1)]) and an associated error term ($\sigma_{R}$[prior: *half‐Cauchy* (*τ* = 0.16)]). We included an intercept adjustment term ($\beta_{RS}$) to allow resighting rates in Florida during the non‐breeding season to differ between individuals banded in NC relative to those banded in VA. We included an intercept‐adjustment term for the band type assigned to an individual ($\beta_{Rb}$) [i.e., (1) service band‐only, (2) early ancillary markers, or (3) plastic field‐readable markers] to account for variation in the probability of resighting an individual during the non‐breeding season associated with the detectability of its band.
(8)

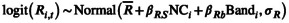

Given the challenges of estimating $R^{\prime }$ (Riecke et al., 2021), we assumed that mortality events occurred, on average, halfway through the non‐breeding season and constrained $R_{t}^{\prime }$ to be equal to $\frac{R_{t}}{2}$.

The observation matrix ($\Omega_{i,j}$) for this model required seven types of observations, which included being (1) seen alive in North Carolina ($\alpha {\hat{p}}_{N}$) and Florida (*R*); (2) seen alive in Virginia ($\alpha {\hat{p}}_{V}$) and Florida (*R*); (3) seen alive in North Carolina ($\alpha {\hat{p}}_{N}$) but missed in Florida (1 − *R*); (4) seen alive in Virginia ($\alpha {\hat{p}}_{V}$) but missed in Florida (1 − *R*); (5) missed in either North Carolina (1 − $\alpha {\hat{p}}_{N}$) or Virginia (1 − $\alpha {\hat{p}}_{V}$) but seen in Florida (*R*); (6) missed in North Carolina (1 − $\alpha {\hat{p}}_{N}$), Virginia (1 − $\alpha {\hat{p}}_{V}$), and Florida (1 − *R*); and (7) recovered and reported dead to the Bird Banding Lab.

Detection on the breeding grounds (*p*) was modeled as the product of its constituent elements; availability (*α*) and detectability ($\hat{p}$). We deviated from the usual nomenclature of ‘true’ detection (i.e., p*; Kendall et al., 1997), as our approach to disentangle *α* and $\hat{p}$ assumed that detection of an individual physically at a breeding colony, $\hat{p}$, was indifferent to an individual's age, and all age‐related variation in *p* was a function of variation in the presence, or availability (*α*) of younger (<7 years) individuals on the breeding grounds. Thus, our estimate of $\hat{p}$ would only be equivalent to p* generated from robust design or spatially explicit models if a series of strong assumptions were realized.

(9) αa=α^a,a≥2a<71,a≥7.

We assumed all seventh year individuals were considered breeding adults and “always” available for detection and younger individuals (2–6) were proportionally less likely to be available (${\hat{\alpha }}_{a}$) on the breeding grounds than the oldest (≥7) age class. The assumption that all ‘breeding‐aged’ adults were available for detection on the breeding grounds each year was most likely violated. Thus, α should be interpreted as the proportion of each sub‐adult age‐class that attempted to breed in a given year relative to the proportion of breeding adults that attempted to breed. Therefore, ${\hat{\alpha }}_{a}$ would represent an estimate for the upper boundary of breeding propensity (hereafter, conditional breeding propensity). Given the life history of ROYT, it was likely that adult breeding propensity was high, which would reduce the magnitude of bias associated with α; however, we do not currently have the data necessary to confirm this assumption. We did not consider temporal or spatial variation in ${\hat{a}}_{a}$ (prior: *beta*[1,1]).

Spatiotemporal variation in detection on the breeding grounds ($\hat{p}$) was modeled with a correlated hierarchical random effects model to account for potential overdispersion and that variation in detection rates on the breeding grounds covaried between NC and VA due to shifts in research interests and activities, where the mean detection rate for each state was drawn from a logistic prior (0,1) and the parameterization of the variance–covariance matrix, $\Sigma_{p}$, was identical to the aforementioned variance–covariance matrices. Similar to both the recovery and resighting models, we included an intercept‐adjustment term for the band type assigned to an individual ($\beta_{pb}$) [i.e., (1) metal‐only, (2) early ancillary markers, or (3) plastic field‐readable markers] to account variation in the probability of detecting an individual during the non‐breeding season associated with the detectability of its band.
(10)

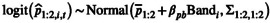




#### 2.2.3 Model building and inference

The overall model was built using the R package *nimble* (de Valpine et al., 2017, 2022). For both the environmental and demographic models, we inferred support for each covariate relationship based on whether 90% of the posterior distribution for each beta coefficient was on the same side of zero as the mean. In the text, we report either the partial correlation (*ρ*, i.e., standardized slope coefficients, $\rho_{x\to y}=\beta_{x\to y}\frac{\sigma_{x}}{\sigma_{y}}$) or unstandardized slope coefficients (β) with the proportion of the posterior distribution on the same side of zero as the mean (*f*). Credible intervals for all parameter estimates are reported in the supplemental material. We ran each model with four chains, a burn‐in period of 50,000 iterations, and model run length of an additional 150,000 iterations with no thinning, which resulted in a posterior distribution based on four chains of 100,000 iterations each. Model convergence was determined by visually inspecting chains with additional validation through the $\hat{R}$ statistic (Brooks & Gelman, 1998). All environmental variables were *z*‐standardized to allow for direct comparisons between correlation coefficients throughout the environmental model. Additionally, as the full model included regressions across multiple link functions (i.e., identity and log–log), and therefore Gaussian and binary responses, we standardized regression coefficients (*β*) generated from binary response outcomes to be equivalent to correlation coefficients (*ρ*) on the linear scale using the latent theoretic approach (Grace et al., 2018) to allow for direct comparisons among all covariances or covariate relationships within a specific model regardless of the link function used. As all regression coefficients were reported in the same scale, indirect associations between two variables were the product of the correlation coefficients of each relevant direct connection in the pathway (e.g., $\rho_{\mathrm{TW}\to \mathrm{DR}}\times \rho_{\mathrm{DR}\to \text{ROYT}}$).

## 3 RESULTS

### 3.1 Associations among environmental variables

**FIGURE 3:**
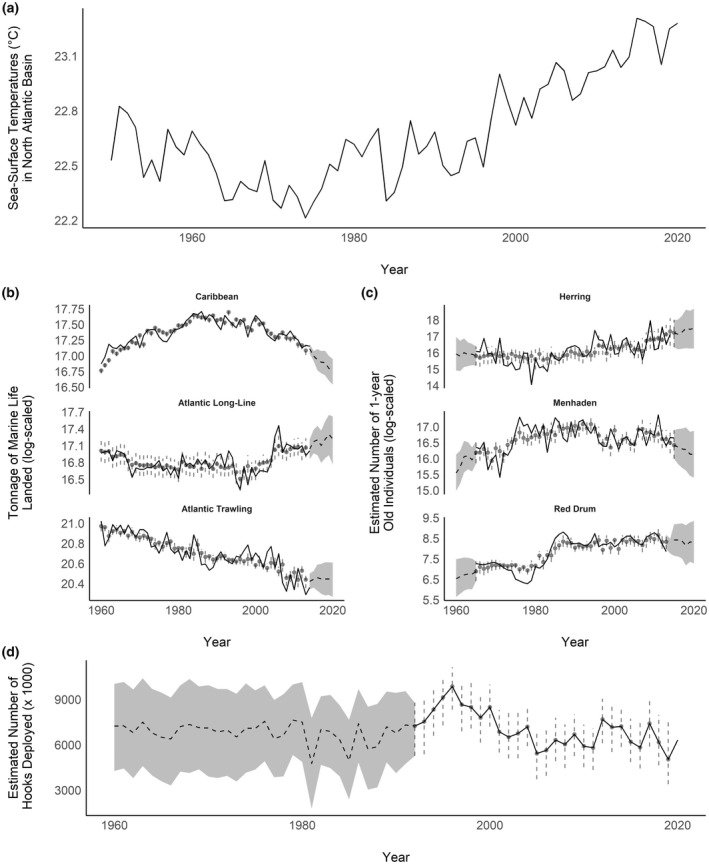
Observed (black lines) and modeled estimates (gray circles with error bars) or predictions (gray ribbons) of the annual variability in (a) average sea‐surface temperature in the North Atlantic; (b) fishery harvest pressures (i.e., tonnage of marine life landed by small‐scale fishing in the Caribbean, commercial long‐line/gill‐net fishing in the North Atlantic, and commercial trawling in the North Atlantic); (c) fishery production (i.e., the estimated number of second‐year Atlantic menhaden, red drum, and herring in the North Atlantic); and (d) the number of hooks deployed (via long‐line fishing) throughout the North Atlantic and Gulf of Mexico over time (1960–2019). Error bars represent 95% Bayesian credible intervals.

The tonnage of fish landed through commercial trawling strongly trended downward ($\rho_{T}$ = −0.91, *f* = 1; Figure 3b, Table S1) with no support for a curvilinear trend ($\rho_{T^{2}}$ = 0.03, *f* = 0.59) over the last 60 years. The tonnage of fish landed throughout the Caribbean increased ($\rho_{T}$ = 0.54, *f* = 1.00) until the early 2000s prior to declining ($\rho_{T^{2}}$ = −0.86, *f* = 1.00) until the end of the study. The tonnage of fish landed by long‐line fishing relatively stable ($\rho_{T}$ = −0.19, *f* = 0.80) until the early 1990s prior to increasing ($\rho_{T^{2}}$ = 0.68, *f* = 1.00) until the end of the study. After accounting for these trends, the residual temporal variation in the tonnage of fish landed by artisanal fishing in the Caribbean and commercial trawling in the North Atlantic (*ρ*_res_ = −0.18, *f =* 0.92; Table S2) were negatively correlated, but fluctuations in long‐line and artisanal fishing in the Caribbean (*ρ*_res_ = −0.09, *f =* 0.73) or trawling in the North Atlantic (*ρ*_res_ = −0.15, *f =* 0.84) were less correlated with each other. SST was associated with reductions in the tonnage of fish landed via artisanal fishing in the Caribbean (*ρ* = −0.14, *f =* 0.98; Table S3), but there was no support for relationships between SST and tonnage of fish landed by commercial trawling or long‐line fishing in the North Atlantic. Counterintuitively, the reported amount of effort (i.e., hooks deployed) associated with long‐line fishing was negatively correlated (*ρ* = −0.40, *f =* 0.96) with the tonnage of fish landed by long‐line fishing, which suggested that increased fishing intensity (i.e., hooks deployed) was associated with a decrease in biomass harvested, and vice versa.

Atlantic menhaden productivity generally increased until the late 1990 s ($\rho_{T}$ = 0.48, *f* = 0.91; Figure 3c) but subsequently declined over the next two decades ($\rho_{T^{2}}$ = −0.99, *f* = 1.00), whereas Atlantic red drum productivity more consistently increased ($\rho_{T}$ = 0.96, *f* = 1.00) over the duration of study with support for a curvilinear pattern ($\rho_{T^{2}}$ = −0.42, *f* = 0.91). Atlantic herring productivity was stable ($\rho_{T}$ = 0.33, *f* = 0.77) with support for an increase in production near the end of the study period ($\rho_{T^{2}}$ = 0.82 *f* = 0.98). After accounting for these trends, the residual temporal variation in red drum and herring productivity were positively correlated ($\rho_{\mathrm{res}}$ = 0.42, *f* = 1.00), but there was no support for any meaningful correlations between Menhaden productivity and productivity of the other species (Table S2). SST was not supported to influence Atlantic menhaden (*ρ* = −0.16, *f =* 0.75; Table S3), red drum (*ρ* = 0.05, *f =* 0.61), or herring (*ρ* = −0.19, *f =* 0.75) production in the western North Atlantic. However, the amount of fish landed via commercial trawling the previous year was associated with a reduction in Atlantic menhaden productivity the following year (*ρ* = −0.14, *f =* 0.98), but we found less support for negative associations between trawling and either Atlantic red drum (*ρ* = −0.13, *f =* 0.80) or herring (*ρ* = −0.29, *f =* 0.86) production. The amount of fish landed via long‐line fishing the previous year was positively associated with Atlantic menhaden (*ρ* = 0.36, *f =* 0.99) but negatively associated with Atlantic herring (*ρ* = −0.26, *f =* 0.90) production the following year; however, there was no support an effect of long‐line fishing on Atlantic red drum (*ρ* = −0.00, *f =* 0.51) productivity.

#### 3.1.1 ROYT observation processes

The average probability that a ROYT was detected during a breeding season (${\bar{p}}_{\mathrm{NC}}=0.001;{\bar{p}}_{\mathrm{VA}}=0.0002$; Figure S1), recovered when dead ($\bar{r}$ = 0.014; Figure S1), or detected alive during a non‐breeding season in Florida ($\bar{R}$ = 0.002; Figure S1) were all extremely low. In addition to have greater breeding season detection probabilities, individuals banded in North Carolina also had greater non‐breeding resighting ($\beta_{R\mathrm{NC}}$ = 0.69; 90%: 0.161–0.77, *f* = 1.00) and recovery ($\beta_{r\mathrm{NC}}$ = 0.08; 90%: 0.03–0.13, *f* = 1.00) rates. The early ancillary banding strategy was not associated with an increase in breeding ($\beta_{p\mathrm{AB}}$ = 0.13; 90%: −0.07–0.33, *f* = 0.86) and non‐breeding detection rates ($\beta_{R\mathrm{AB}}$ = 0.10; 90%: −0.09–0.29, *f* = 0.80), but was an associated with an increase in recovery rates ($\beta_{r\mathrm{AB}}$ = 0.29; 90%: 0.14–0.44, *f* = 1.00), relative to individuals fit with only a service band. During the last few years of the study, individuals fit with plastic field‐readable bands were approximately 1.43 times more likely to be recovered ($\beta_{r\mathrm{PFR}}$ = 0.36; 90%: 0.16–0.56, *f* = 1.00), 9.39 times more likely to be observed during the non‐breeding season ($\beta_{R\mathrm{PFR}}$ = 2.24; 90%: 2.04–2.44, *f* = 1.00), and 4722 times more likely to be observed during the breeding season ($\beta_{p\mathrm{PFR}}$ = 8.47; 95%: 6.48–10.43, *f* = 1.00) relative to individuals with only a service band (Table S4).

Of the three types of reencounters, recovery rates (*r*) were the most likely and least temporally variable (*σ*_*r*_ = 0.28; Figure S1). Detection probabilities in Florida during the non‐breeding season exhibited substantially more among‐year variation (*σ*_*R*_ = 1.59) and following the creation of BBL's band reporting website (circa 1999) increased, on average, by an order of magnitude (${\bar{R}}_{\mathrm{pre}1999}$ = 0.001; ${\bar{R}}_{\text{post}1999}$ = 0.012; Figure S1). Resightings on the breeding grounds exhibited the greatest temporal variability ($\sigma_{\mathrm{NC}}$ = 2.23; $\sigma_{\mathrm{VA}}$ = 4.82) and were effectively only non‐zero during the time periods in which research on the breeding grounds was active (NC and VA: 1970–1985; VA: 2017–2020).

#### 3.1.2 General patterns in ROYT demographic rates

**FIGURE 4:**
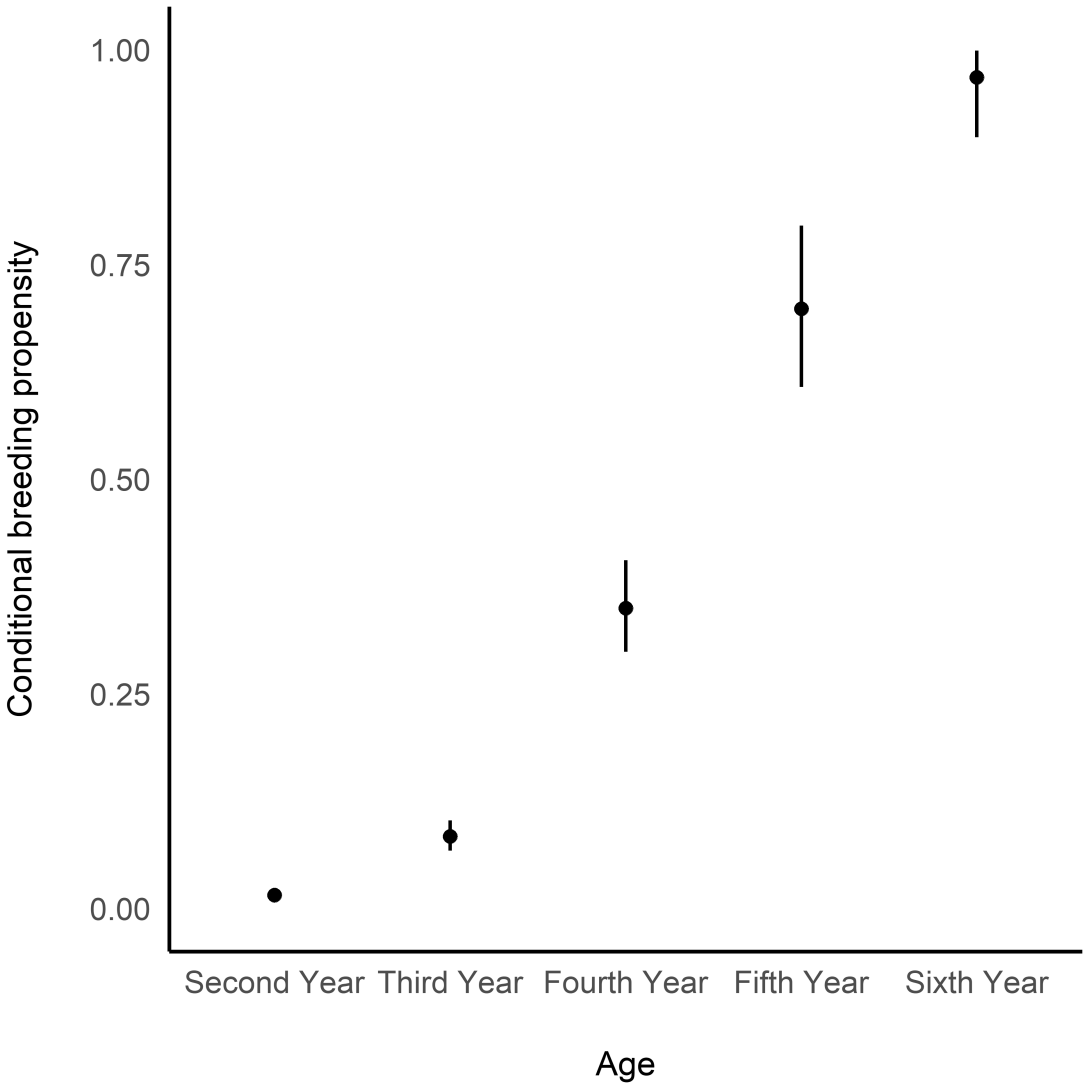
Average age‐specific conditional breeding propensities, or the proportion of each age‐class that was available for detection on the breeding grounds relative to the average breeding adult (7+ years), for Royal terns banded in North Carolina or Virginia from 1961 to 2021. No second‐year individuals were ever encountered on the breeding grounds. Error bars represent 95% Bayesian credible intervals.

Fidelity to the mid‐Atlantic breeding population ($\bar{F}$ = 0.998; 90%: 0.996–0.999) and to each state's breeding population ($\bar{\psi }$ = 0.984; 90%: 0.983–0.985) were both high. Conditional breeding propensities (${\hat{a}}_{a}$) were substantially different among the early age‐groups (Figure 4) as only 1% of 2‐year olds (${\hat{a}}_{2}$ = 0.01; 90%: 0.01–0.02); 8% 3‐year old (${\hat{a}}_{3}$ = 0.08; 90%: 0.07–0.10), 35% of 4‐year old (${\hat{a}}_{4}$ = 0.35; 90%: 0.30–0.39), and 69% of 5‐year old (${\hat{a}}_{5}$ = 0.70; 90%: 0.62–0.78) individuals were available for detection on the breeding grounds relative to the average “breeding” aged adult. Although we limited the number of age‐groups allowed to be different from “breeding” aged adults to the ages between 2 and 6, conditional breeding propensity was approaching 1.0 by an individual's sixth year (${\hat{a}}_{6}$ = 0.97; 90%: 0.93–1.00), which suggested that any additional age classes considered would not be distinguishable from an adult.

**FIGURE 5:**
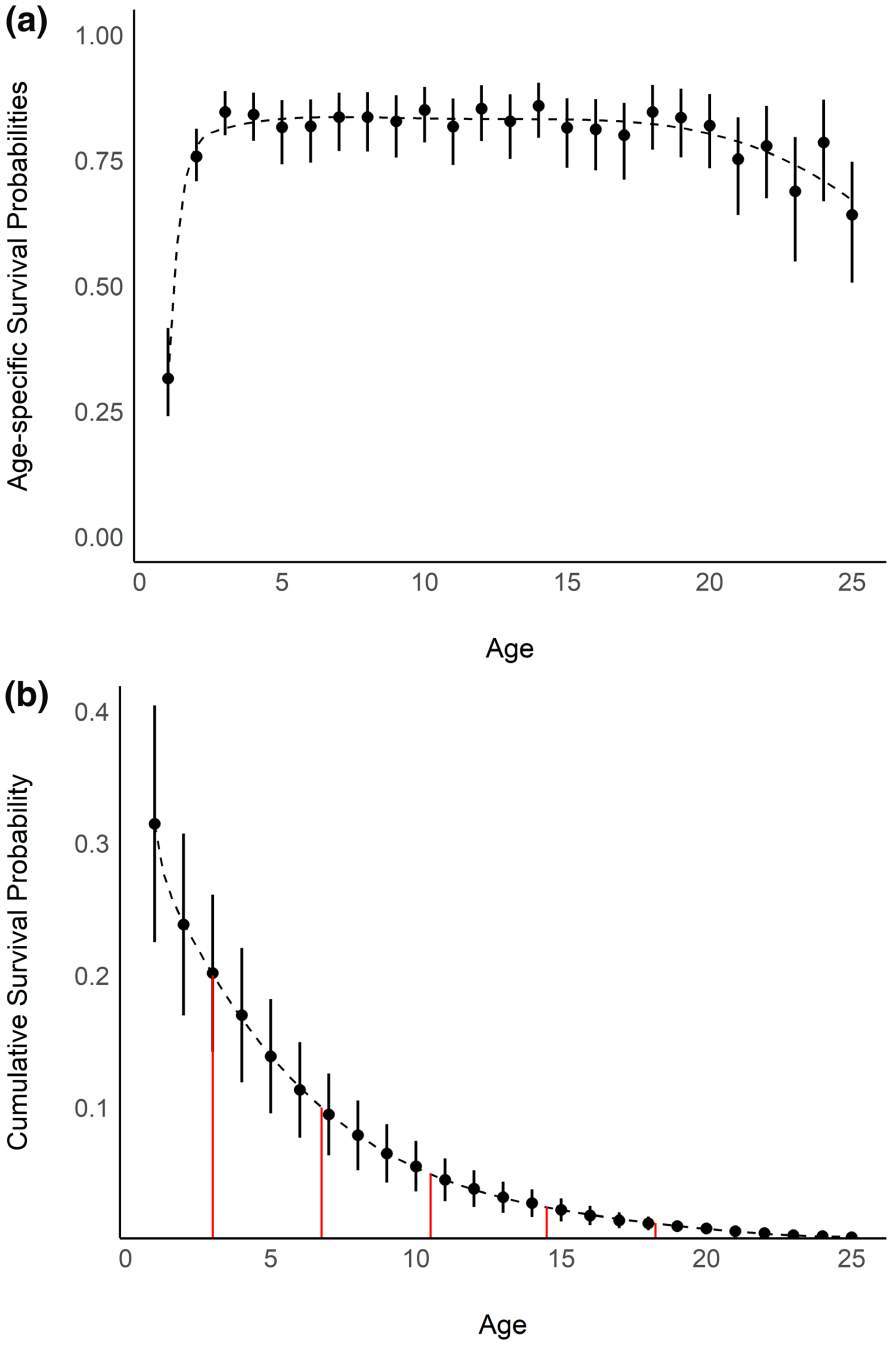
(a) Average age‐specific annual survival probabilities and the corresponding (b) cumulative probability of surviving from approximately 3 weeks of age until a specific age. Dashed lines represent a smooth regression line to assist with visualizing trends. Red vertical lines (in b) indicate the ages in which (from left to right) 20%, 10%, 5%, 2.5%, and 1.25% of the banded cohort were predicted to remain alive. Error bars represent 95% Bayesian credible intervals.

Most ROYT mortality occurred during the first year of life (${\bar{S}}_{\mathrm{FY}}:$ 0.31; 90%: 0.24–0.38; Figure 5a), with survival by the third year (${\bar{S}}_{\mathrm{TY}}:$ 0.85, 90%: 0.80–0.88) being similar to that of breeding‐aged adults (${\bar{S}}_{\mathrm{AD}}:$ 0.81, 90%: 0.78–0.84). Shifts in survival related to advanced age or senescence were subtle but possibly occurred around the age of 20 (Figure 5a). However, as only approximately 1.25% of all banded individuals survived until this age (Figure 5b) and we did not control for individual heterogeneity among individuals (Cam et al., 2002), inference was limited due to sparseness in both the number of individuals that remained in the population and low encounter/recovery rates.

#### 3.1.3 Associations between environmental features and ROYT demography

**FIGURE 6:**
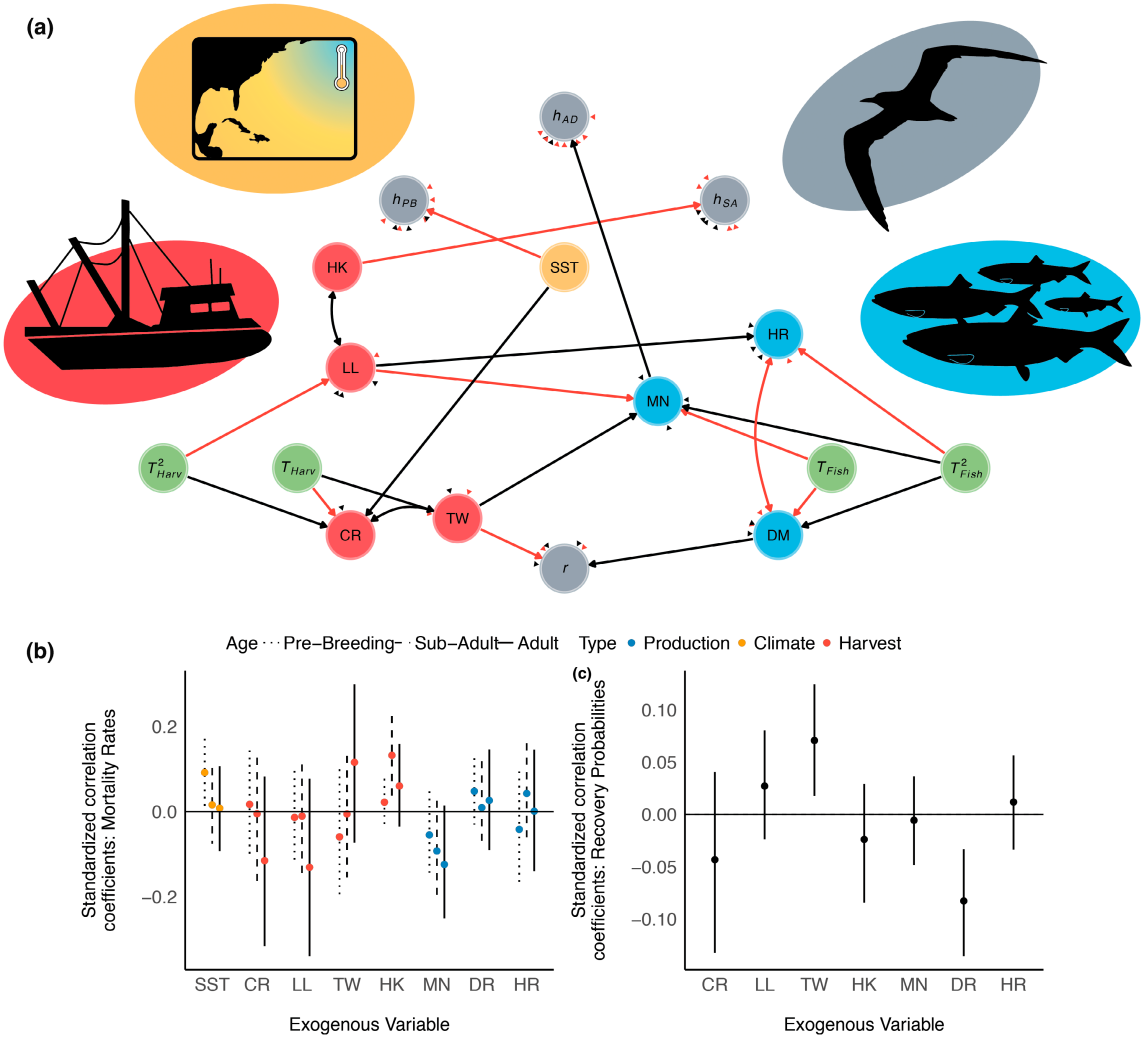
(a) The supported model structure and corresponding effect sizes (b, c) describing the direct (i.e., node to node) and indirect (i.e., node through node) effects of (red: Positive, black: Negative) among various sources of fishery harvest pressure (red circles) and fish production (blue circles), as well as sea‐surface temperature (yellow circle) and time trends (green circles) on pre‐breeding (PB), subadult (SA), and adult (AD) Royal tern (b) mortality and (c) recovery probabilities. (a) Correlations between variables drawn from a multivariate normal distribution were represented with a double‐headed arrow. Unidirectional associations were represented as a single‐headed arrow. Lines between nodes indicated that at least 90% of the highest posterior distribution interval [HPDI] for the parameter coefficient was on the same side of zero as the median. Arrow heads with no lines represented unsupported (HPDI <90%) associations between environmental variables or demographic processes. Confidence intervals depict 90% HPDI. Linear (*T*) and quadratic (*T* ^2^) time trends for associations with fishery harvest pressure/intensity (Harv) and fishery production (Fish) were independent from one another. Exogenous variables associated with fishery harvest pressure included the estimated landings via commercial long‐line fishing (LL) and trawling (TW) throughout the Atlantic Coast of North America and artisanal fishing throughout the Caribbean (CR), as well as the estimated number of hooks deployed via long‐ling fishing throughout the North Atlantic and Gulf of Mexico (HK). Variables associated with fishery production included the estimated number of 1‐year‐old Atlantic herring (HR), Atlantic menhaden (MN) and red drum (DM). Sea‐surface temperatures (SSTs) were used to model shifts in climate conditions. Each exogenous variable was allowed to influence age‐class specific (pre‐breeding (PB), subadult (SA), and adult (AD)) mortality risks (*h*) for Royal terns; however, only fishery harvest and production variables were considered to influence Royal tern recovery probabilities (*r*).

ROYT recovery probabilities were negatively correlated with Atlantic red drum productivity (*ρ* = −0.08, *f =* 0.99) but positively correlated with landings by commercial trawling in the North Atlantic (*ρ* = 0.07, *f =* 0.97). All other sources of variation in fishery harvest pressure or fish production were not supported to influence recovery probabilities (Table S4; Figure 6). Atlantic menhaden production was negatively associated with adult ($\rho_{\mathrm{AD}}$ = −0.13, *f =* 0.91) and to a lesser extent sub‐adult ($\rho_{\mathrm{SA}}$ = −0.09, *f =* 0.88) mortality (Table S5; Figure 6). However, Atlantic herring and red drum production were not supported to influence age‐specific mortality (Table S5). The number of hooks deployed via long‐line fishing in the North Atlantic was positively associated with the risk of sub‐adult mortality ($\rho_{\mathrm{SA}}$ = 0.13, *f =* 0.97). Although the tonnage landed by long‐line fishing or commercial trawling in the North Atlantic, as well as artisanal fishing throughout the Caribbean, were not supported to directly influence the risk of ROYT mortality (Table S5), commercial trawling was indirectly associated with an increased risk of adult mortality ($\rho_{\mathrm{Trw}\to \mathrm{Men}}$ = 0.05, *f =* 0.90) as a function of trawling being negatively associated with menhaden production (Figure S2). In contrast, long‐line fishing was indirectly associated a decreased risk of sub‐adult ($\rho_{\mathrm{LL}\to \mathrm{HK}}$ = −0.06, *f =* 0.95) and adult ($\rho_{\mathrm{LL}\to \mathrm{Men}}$ = −0.05, *f =* 0.90) mortality as a function of a (1) negative association between long‐line fishing and the number of hooks deployed in the North Atlantic; and (2) a positive association between long‐line fishing and menhaden production. Lastly, SST was positively associated with pre‐breeding mortality risk ($\rho_{\mathrm{PB}}$ = 0.09, *f =* 0.95), which suggested that warmer conditions were associated with lower survivorship during the first 2 years of life.

**FIGURE 7:**
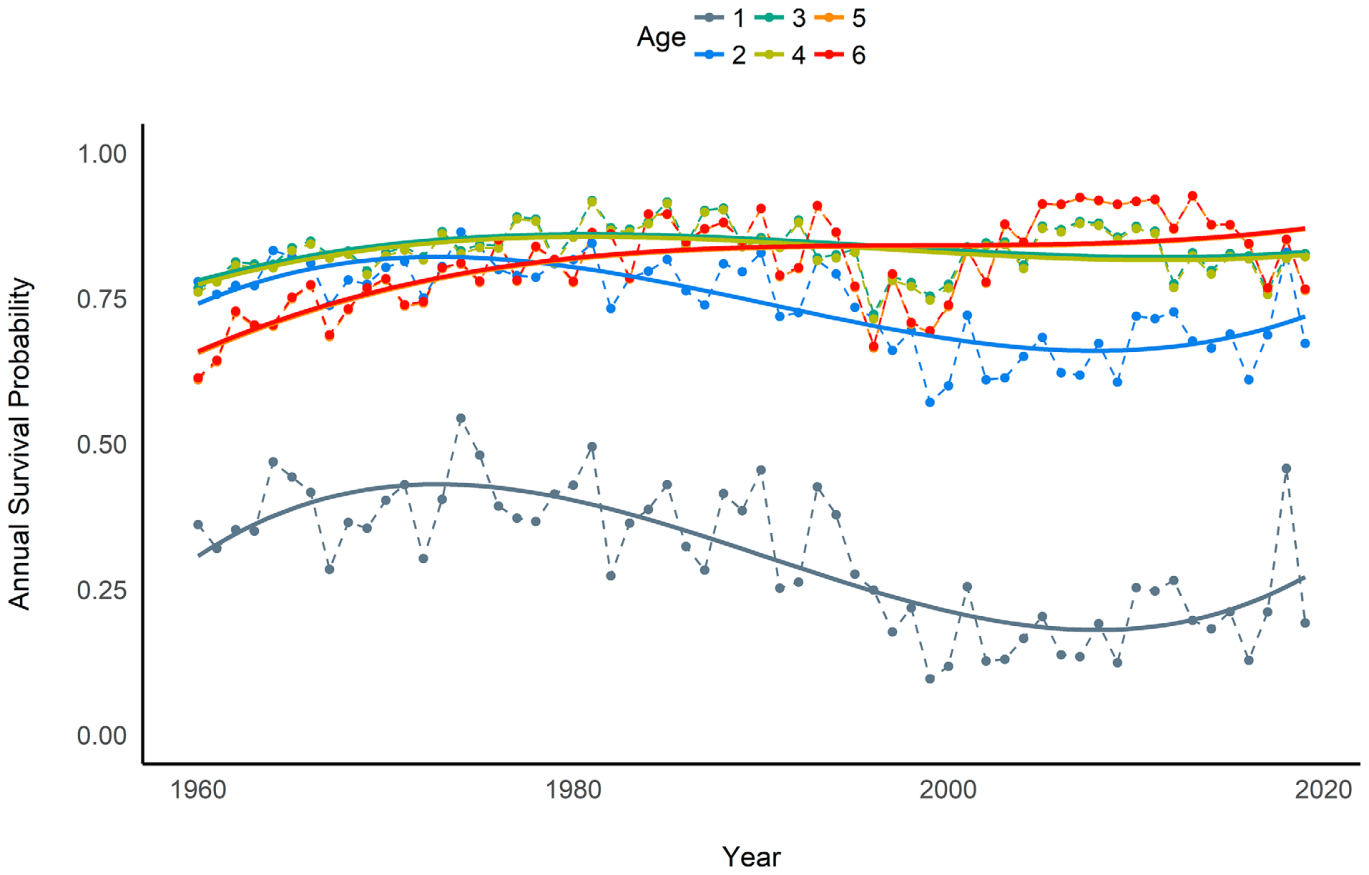
Estimated Royal tern annual survival probabilities during the first 6 years of life from 1961 to 2021. Smoothed solid lines represented a third‐order spline function fit to the modeled survival estimates for each age and were not used to estimate the specific parameters.

**FIGURE 8:**
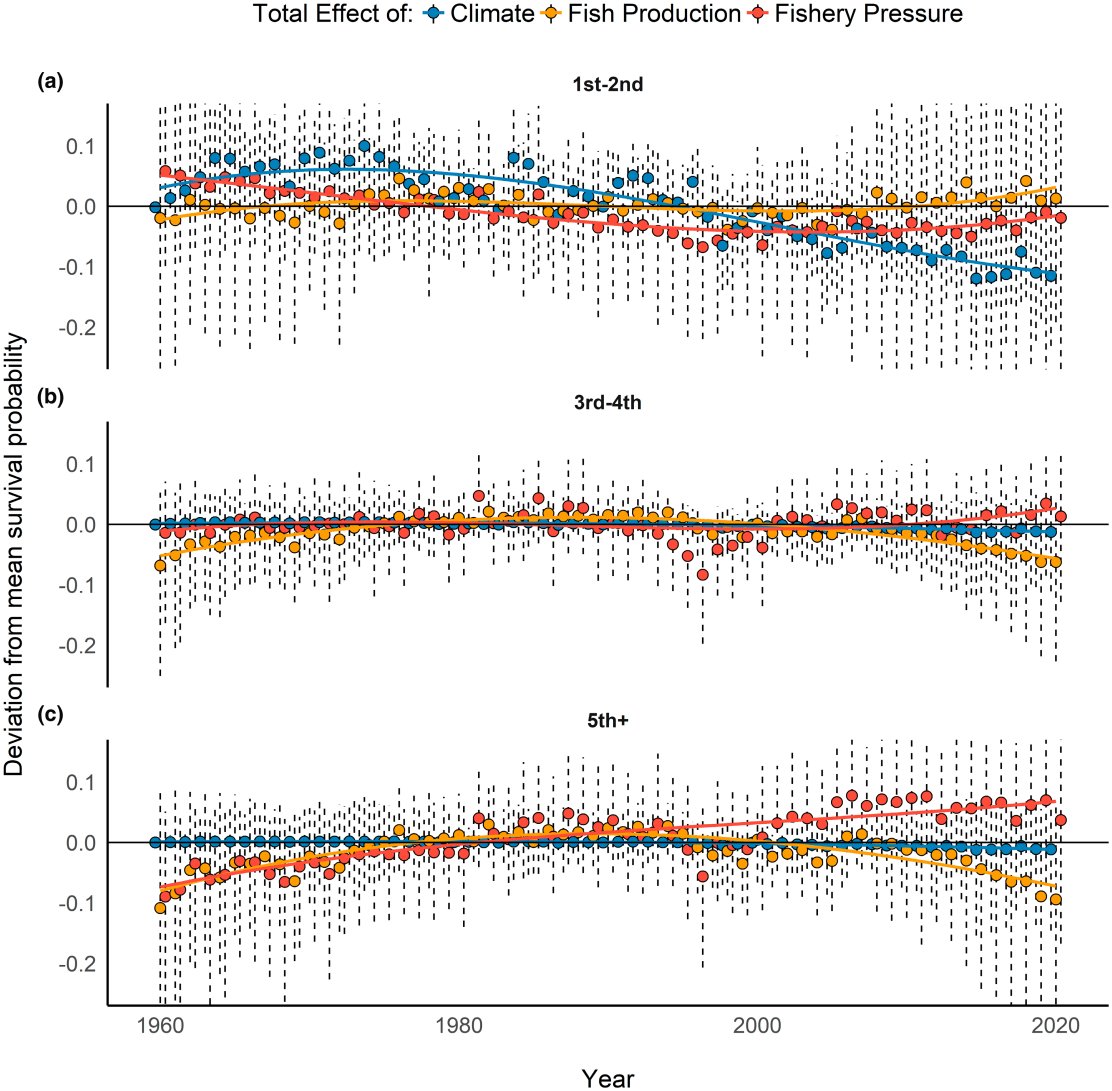
The total effects of variation in sea‐surface temperature (blue), fish production (yellow: relative abundance of 1‐year‐old Atlantic menhaden, herring, and red drum), and fishery harvest pressure (red: Landings via commercial long‐line and trawling in the North Atlantic or artisanal fishing in the Caribbean; hooks deployed in North Atlantic/Gulf of Mexico) on deviations in age‐specific (a: first and second year, b: third and fourth year, c: fifth year and older) survival from the mean from 1961 to 2021. Error bars represent 95% Bayesian credible intervals. Smoothed solid lines represented a third‐order spline function fit to the model estimates for each age‐class and were not used to estimate the specific parameters.

**FIGURE 9:**
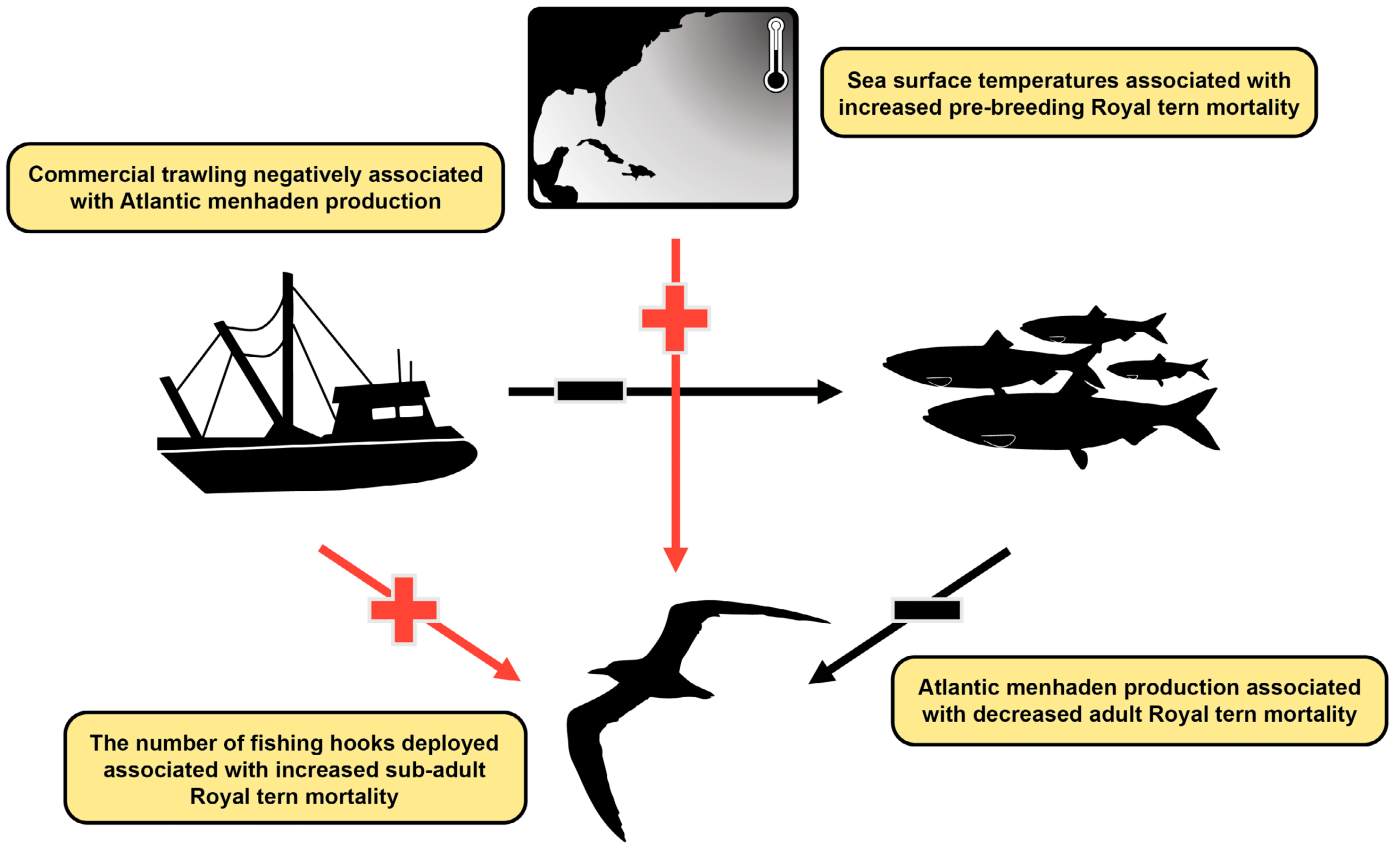
Simplified path diagram describing the primary pathways in which environmental variation (i.e., sea‐surface temperatures in the North Atlantic, fishery pressure, and fish production) was supported to indirectly and directly influence age‐specific Royal tern mortality.

Due to the amount of variation in environmental conditions (Figure 3) and their impact on ROYT mortality (Figure 6), survival rates for ROYT fluctuated among years and between age classes from 1961 to 2019 (Figure 7). Of note, pre‐breeding and adult ROYT survivorship were mostly influenced by bottom‐up mechanisms (e.g., climate conditions and food resources), whereas sub‐adults were more impacted by top‐down constraints (i.e., entanglement risk; Figure 9). Although all age‐classes experienced a decline in survival during the 1990s (Figure 7), pre‐breeding survival had been trending downwards for a decade prior, which temporally coincided with a period of consistently increasing SSTs that continued to the end of the study period (Figures 3a and 8). Ultimately, the observed trends in environmental conditions has resulted in a 55% decline in survivorship from 3 weeks of age until the age at which over half of the surviving cohort has recruited into the breeding population (~5 years old) between the first (1961–1990: ${\bar{S}}_{1-5}$ = 0.18) and second (1991–2017: ${\bar{S}}_{1-5}$ = 0.081) halves of the study, which was primarily driven by the decline in pre‐breeding survival associated with increased SST.

## 4 DISCUSSION

Here, we demonstrated that climate, fishery pressure, and fishery production were functionally connected and, together, have codetermined age‐specific survival of ROYT since the 1960s. Specifically, we found that (1) both top‐down and bottom‐up forces have influenced ROYT population dynamics (Figures 7 and 9), (2) the general trajectories of the environmental features associated with these competing regulatory constraints have trended in opposite directions (Figure 8), and (3) the overall outcome of these shifting environmental trajectories has disproportionately impacted the youngest age‐classes of ROYT (Figure 8). Combined, these phenomena have resulted in the general trends in age‐specific survivorship patterns that substantially deviate from one another over a 60‐year period. This deviation in survivorship among age‐classes (Figure 8) was predominantly related to (1) younger individuals being more sensitive to variation in SST relative to older individuals, (2) older individuals being more demographically responsive to shifts in fishery pressures relative to younger individuals, and (3) opposing trends in SSTs and landings via commercial fishing [e.g., warming seas (Figure 3a); reductions in landings by commercial trawling (Figure 3b) and hooks deployed] over the last 60 years in the North Atlantic. Although sub‐adult survival was generally not meaningfully different from adult survival for most of the study period, there was evidence that these two demographic traits have recently diverged in response to age‐specific sensitivities to life‐history trade‐offs or environmental conditions.

Previous work has consistently demonstrated that increased SSTs result in reductions in foraging success (Carroll et al., 2016; Divoky et al., 2021; Guinet et al., 1998; Sandvik et al., 2008; Thayer et al., 2008), reproductive success (Frederiksen et al., 2007; Peck et al., 2004; Smithers et al., 2003), survival (Sandvik et al., 2005) and regional abundance (Irons et al., 2008; Veit et al., 1996) of seabird populations across the globe. Likewise, incidental bycatch is considered to be a substantial source of seabird mortality (Anderson et al., 2011; Croxall et al., 2012) that may vary among age classes (Gianuca et al., 2017). However, the actual demographic impacts of fisheries on seabird survival are rarely determined (but see Genovart et al., 2017). Here, we expand on these results by simultaneously demonstrating the relative importance of fishery effort, fish production, and climate conditions on age‐specific survival, while demonstrating how these processes were temporally connected to one another. Additionally, we were able to demonstrate that relative importance of top‐down and bottom‐up pressures on demographic rates vary across time and that bottom‐up processes can affect top‐down pressures. These results highlight the importance of (1) long‐term demographic data for understanding the forces regulating populations and (2) the use of network‐based inferential models as they provide the ability to discern between multiple competing, and potentially correlated hypotheses.

Commercial fishing has often been directly linked to seabird mortality (Anderson et al., 2011; Buckley & Buckley, 1974; Tasker, 2000; Watkins et al., 2008) with population‐ and species‐level consequences. In addition to mortality associated with entanglement with fishing gear, there are multiple other direct (e.g., disturbance) and indirect (e.g., stock depletion [−], access to discards [+]) mechanisms that may negatively or positively influence seabird population dynamics (Tasker, 2000). Here, our approach attempted to disentangle the top‐down (e.g., entanglement) and bottom‐up (e.g., fish removal) impacts of commercial fishing. Specifically, we found that the industrial trawling was more likely indirectly associated with increased adult ROYT mortality by reducing Atlantic menhaden production, a primary component of ROYT diet during the breeding season (Wambach & Emslie, 2003), whereas the number of hooks deployed by long‐line fishing was directly linked with increased sub‐adult mortality. Additionally, the amount of biomass landed and the amount of gear used in a given year by the long‐line fishing industry were negatively correlated with one another, which suggested (1) total landings may not be an ideal index of entanglement risk and (2) quota‐based regulatory frameworks may result in increased risk to seabirds due to an increase in gear deployed during years of reduced fish stocks. Likewise, certain measures of harvest pressure (i.e., landings by long‐line fishing) were potentially confounded with ecosystem health (i.e., Atlantic menhaden production), whereas others were likely describing fluctuations in fishery exploitation (i.e., commercial trawling) or entanglement risk (i.e., number of hooks). Ultimately, commercial fishing along the Atlantic Coast is managed to maximize the sustainability of fish populations while limiting broader ecological impacts (Kaiser et al., 2016). However, the extent to which fishing is considered to be sustainable varies spatially and among fisheries (Amoroso et al., 2018). As such, if the indirect association between ROYT survival and trawling along the Atlantic Coast were, in fact, related to shifts in fish distributions, abundance, or availability, our results would suggest the working definition of a sustainable fishery is ecologically misguided in the lens of seabird conservation.

Although our retrospective analysis cannot determine the specific mechanism(s) linking SST and ROYT mortality, we speculate that climate‐related shifts in foraging efficacy or prey abundance or distributions are likely explanations. Furthermore, as first‐year individuals are completely dependent on their parents for food through the offspring's first winter (Ashmole & Tovar, 1968), reduced foraging success would likely negatively impact the entire family unit. However, this downward shift in pre‐breeding survivorship from 2000 to 2020 temporally coincided with a decreased risk in adult mortality associated with commercial fishing. Given the energy investment and risk associated with parental care in this species (Ashmole & Tovar, 1968), the divergence in survival rates between pre‐breeding and adult individuals may be partially confounded with an intrinsic life history trade‐off, where adult survival, on average, increased due to a decrease in population‐level paternal obligations as a function of increased mortality of first‐year individuals (Stearns, 2000). Regardless of the specific mechanism, the observed impact of SST on survival and the observed increase in SST over the last century indicates ROYT may experience continued reductions in population size in the near future (Emslie et al., 2009) due to a failure to recruit offspring into the breeding population.

### 4.1 Conservation implications

Our results indicate that climate change should continue to be considered a major threat to the population persistence of the mid‐Atlantic ROYT metapopulation. Although this study primarily focused on the drivers of age‐specific survival, ROYT within the mid‐Atlantic face additional climate and anthropogenic threats to their population persistence. In North Carolina, reductions in dredge spoil material supplied to seabird islands by the U.S. Army Corps of Engineers over the last two decades in conjunction with unmitigated widening of shipping channels allowed vegetation to encroach and exacerbated erosion, reducing the amount of the available habitat for individuals to breed (Emslie et al., 2009; USACE, 2020). In the Chesapeake Bay region of Maryland and Virginia, most of the islands historically associated with ROYT colonies during the last century have either eroded, been colonized by mammalian nest predators, or degraded due to vegetation encroachment and sea‐level rise (Brinker et al., 2007). This has resulted in a novel conservation crisis as the breeding ROYT population in Maryland approaches extirpation, and the vast majority of ROYT that breed in Virginia have colonized (ca. 2006) a man‐made island connected to Interstate 64 between Hampton and Norfolk, VA. As both the regional and within‐region site fidelity of ROYT were, on average, extremely high during the length of this study despite substantial shifts in breeding habitat composition or quality throughout the region, conservation actions focused on establishing novel breeding habitats outside this region may not be successful. Ultimately, as survival from 3 weeks of age until recruitment has declined by approximately 55% between the first (1961–1990) and second (1991–2017) halves of the study period, any additional threat to the species' seasonal habitat may have an outsized impact on population persistence. Thus, given that sea‐level rise and the associated loss of coastal areas within the region are inevitable (Wu et al., 2009), additional research is needed to demonstrate the relative contributions of survival and habitat‐related reproductive output to population persistence and what this means for the conservation of the mid‐Atlantic metapopulation in the future.

## AUTHOR CONTRIBUTIONS

Daniel Gibson, Thomas V. Riecke, Daniel H. Catlin, Kelsi L. Hunt, Sarah M. Karpanty, and James D. Fraser designed the study. Daniel Gibson, Thomas V. Riecke, and David N. Koons contributed to scripts. Daniel Gibson analyzed the data and wrote the manuscript. Daniel Gibson, Thomas V. Riecke, Kelsi L. Hunt, Chelsea E. Weithman, Sarah M. Karpanty, and James D. Fraser edited and improved the manuscript.

## CONFLICT OF INTEREST

The authors declare that they have no conflicts of interests.

## Supporting information


Appendix S1

**Figure S2**. The estimated indirect effects of environmental variables, and the directional pathway by which the variable was associated with pre‐breeding (gray), subadult (green), and adult (red) Royal tern mortality. Confidence intervals depict 90% HPDI.

## Data Availability

The model code, mark‐recapture, and environmental data used to support the results presented in this study are openly available at Dryad at https://doi.org/10.5061/dryad.mcvdnck3n.
