# Supplementary material for: Climate change and commercial fishing practices codetermine survival of a long‐lived seabird
Source: Glob Chang Biol. 2022 Oct 22;29(2):324–40. doi: 10.1111/gcb.16482 (PMC10092490; doi:10.1111/gcb.16482)

**SUPPLEMENTAL MATERIAL**

The process matrix ($\Psi_{i,j}$; eqn S1) for the multistate Barker model included three living states (1: alive, in the NC breeding population; 2: alive, in the VA breeding population; and 3) alive, but permanently emigrated from the mid-Atlantic (NC and VA) metapopulation during or prior the most recent timestep) and three dead states (4: recovered and reported as dead during most recent timestep; 5) died during the most recent timestep, but was not resighted; and 6) died during a previous timestep). Here, transitions among the living states were a function of survival (S) and the two movement parameters (*F* and ψ), where *F* represented the proportion of each surviving breeding population that did not permanently leave the mid-Atlantic metapopulation between two breeding seasons and ψ represented the proportion of each surviving breeding population that remained in the state (NC or VA) that they were associated during the previous breeding season. Transitions from the living states to the dead states were a function of the probability of mortality during a specific timestep (1-S) and the probabilities of being recovered during that timestep (*r*) or being encountered alive during, but prior to death, the non-breeding season an individual ultimately died (*R’*). Transitions among dead states were unidirectional (i.e., from each dead state to the ‘previous dead’ or terminal dead state) and fixed to 1.0.

|  | $\Psi_{i,j}=\left\vert\begin{matrix} SF\psi& SF\left( 1-\psi\right) & S\left( 1-F \right) & \left( 1-S \right)r & \left( 1-S \right)R^{'}\left( 1-r \right) & (1-S){(1-R}^{'})(1-r) \\ SF\left( 1-\psi\right) & SF\psi& S\left( 1-F \right) & \left( 1-S \right)r & \left( 1-S \right)R^{'}\left( 1-r \right) & (1-S){(1-R}^{'})(1-r) \\ 0 & 0 & S & \left( 1-S \right)r & \left( 1-S \right)R^{'}\left( 1-r \right) & (1-S){(1-R}^{'})(1-r) \\ 0 & 0 & 0 & 0 & 0 & 1 \\ 0 & 0 & 0 & 0 & 0 & 1 \\ 0 & 0 & 0 & 0 & 0 & 1 \end{matrix} \right\vert$ | (S1) |
| --- | --- | --- |

The observation matrix ($\Omega_{i,j}$, eqn S2) for this model required seven types of observations, which included being 1) seen alive in North Carolina ($\alpha\hat{p}_{N}$) and Florida (*R*); 2) seen alive in Virginia ($\alpha\hat{p}_{V}$) and Florida (*R*); 3) seen alive in North Carolina ($\alpha\hat{p}_{N}$) but missed in Florida (1-*R*); 4) seen alive in Virginia ($\alpha\hat{p}_{V}$) but missed in Florida (1-*R*); 5) missed in either North Carolina (1-$\alpha\hat{p}_{N}$) or Virginia (1-$\alpha\hat{p}_{V}$) but seen in Florida (*R*); 6) missed in North Carolina (1-$\alpha\hat{p}_{N}$), Virginia (1-$\alpha\hat{p}_{V}$), and Florida (1-*R*); and 7) recovered and reported dead to the Bird Banding Lab.

|  | $\Omega_{i,j}=\left\vert\begin{matrix} \alpha\hat{p}_{N}R & 0 & \alpha\hat{p}_{N}\left( 1-R \right) & 0 & \left( 1-\alpha\hat{p}_{N} \right)R & \left( 1-\alpha\hat{p}_{N} \right)\left( 1-R \right) & 0 \\ 0 & \alpha\hat{p}_{V}R & 0 & \alpha\hat{p}_{V}\left( 1-R \right) & \left( 1-\alpha\hat{p}_{V} \right)R & \left( 1-\alpha\hat{p}_{V} \right)\left( 1-R \right) & 0 \\ 0 & 0 & 0 & 0 & R & (1-R) & 0 \\ 0 & 0 & 0 & 0 & 0 & 0 & 1 \\ 0 & 0 & 0 & 0 & 1 & 0 & 0 \\ 0 & 0 & 0 & 0 & 0 & 1 & 0 \end{matrix} \right\vert$ | (S2) |
| --- | --- | --- |

**SUPPLEMENTARY TABLES**

**Supplemental Table 1.** Estimated standardized slope coefficients (ρ), their associated uncertainty (90% Highest Posterior Density Intervals), and the proportion of the posterior distribution on the same side of zero as the mean (*f*) describing the various temporal trends (Linear, Autoregressive) to determine shifts in environmental conditions in the North Atlantic from 1960–2019. Parameter estimates with 90% of the posterior distributions on the same side of zero as the mean are presented in black text. $\hat{\boldsymbol{R}}$ values less than 1.10 were considered to be indicative of parameter convergence.

.

| **Parameter Type** | **Responding Variable** | **Slope Coefficient (**ρ**)** | **90% HPDI** | ***f*** | $\hat{\boldsymbol{R}}$ |
| --- | --- | --- | --- | --- | --- |
| Linear Trend | Caribbean | 0.54 | 0.42 – 0.67 | 1.00 | 1.00 |
| Linear Trend | Long-line | -0.19 | -0.58 – 0.17 | 0.80 | 1.01 |
| Linear Trend | Trawling | -0.91 | -1.16 – -0.68 | 1.00 | 1.02 |
| Linear Trend | Menhaden | 0.48 | -0.13 – 1.09 | 0.91 | 1.03 |
| Linear Trend | Red drum | 0.96 | 0.55 – 1.47 | 1.00 | 1.01 |
| Linear Trend | Herring | 0.33 | -0.38 – 1.05 | 0.77 | 1.02 |
| Quadratic Trend | Caribbean | -0.86 | -0.98 – -0.74 | 1.00 | 1.00 |
| Quadratic Trend | Long-line | 0.68 | 0.32 – 1.02 | 1.00 | 1.01 |
| Quadratic Trend | Trawling | 0.03 | -0.21 – 0.26 | 0.58 | 1.02 |
| Quadratic Trend | Menhaden | -0.99 | -1.49 – -0.66 | 1.00 | 1.00 |
| Quadratic Trend | Red drum | -0.42 | -0.94 – 0.10 | 0.91 | 1.02 |
| Quadratic Trend | Herring | 0.82 | 0.29 – 1.46 | 0.98 | 1.01 |
| Auto-Regressive (1) | Caribbean | 0.60 | 0.45 – 0.74 | 1.00 | 1.00 |
| Auto-Regressive (1) | Long-line | 0.17 | -0.11 – 0.47 | 0.86 | 1.01 |
| Auto-Regressive (1) | Trawling | 0.34 | 0.15 – 0.54 | 1.00 | 1.00 |
| Auto-Regressive (1) | Hooks | 0.47 | -0.05 – 1.00 | 0.90 | 1.03 |
| Auto-Regressive (1) | Menhaden | 0.22 | 0.01 – 0.44 | 0.95 | 1.01 |
| Auto-Regressive (1) | Red drum | 0.70 | 0.55 – 0.85 | 1.00 | 1.02 |
| Auto-Regressive (1) | Herring | 0.08 | -0.18 – 0.32 | 0.68 | 1.02 |

**Supplemental Table 2.** Estimated residual (i.e., with explanatory variables; ρ_r_), their associated error (90% Highest Posterior Density Intervals), and the proportion of each posterior distribution on the same side of zero as the mean (*f_r_*) in the temporal error in fishery harvest/pressure (Artisanal fishing in Caribbean, Long-line or Trawling in the Atlantic, number of hooks deployed in Atlantic/Gulf of Mexico), fish production (Atlantic menhaden, red drum, and herring 1^st^ year abundance), or age-specific (Pre-Breeding [First & Second Year], Sub-adult [Third& Fourth Year], and Adult [5+ year] Royal tern mortality from 1961–2021 [climate] or 1961–2021 [all other variables]. Parameter estimates with 90% of the posterior distributions on the same side of zero as the mean are presented in black text. $\hat{\boldsymbol{R}}$ values less than 1.10 were considered to be indicative of parameter convergence.

| **Explanatory Variable** | **Correlated Variable** | **Residual Correlation Coefficient (ρ*_r_*)** | **90% HPDI** | ***f_r_*** | $\hat{\boldsymbol{R}}$ |
| --- | --- | --- | --- | --- | --- |
| Menhaden | Red drum | -0.12 | -0.38 – 0.13 | 0.79 | 1.00 |
| Menhaden | Herring | -0.03 | -0.28 – 0.22 | 0.57 | 1.00 |
| Red drum | Herring | 0.41 | 0.20 – 0.62 | 1.00 | 1.00 |
| Hooks | Long-Line | -0.40 | -0.77 – -0.05 | 0.96 | 1.03 |
| Trawling | Long-Line | -0.15 | -0.39 – 0.09 | 0.84 | 1.01 |
| Long-Line | Caribbean | -0.09 | -0.34 – 0.15 | 0.73 | 1.00 |
| Caribbean | Trawling | -0.18 | -0.38 – 0.03 | 0.92 | 1.00 |
| Pre-Breeding | Sub-Adult | 0.06 | -0.49 – 0.62 | 0.61 | 1.09 |
| Pre-Breeding | Adult | 0.15 | -0.21 – 0.51 | 0.76 | 1.01 |
| Sub-Adult | Adult | 0.18 | -0.44 – 0.79 | 0.72 | 1.02 |

**Supplemental Table 3.** Estimated standardized slope coefficients (ρ), their associated error (90% Highest Posterior Density Intervals), and the proportion of the posterior distribution on the same side of zero as the mean (*f*) describing the associations between variation in climate (SST) or fishery pressure in the North Atlantic (Trawling and Long-line fishing) and variation in fish production (Atlantic menhaden, red drum, and herring 1^st^ year abundance) or fishery pressure throughout the Caribbean or North Atlantic from 1961–2021. Parameter estimates with 90% of the posterior distributions on the same side of zero as the mean are presented in black text. $\hat{\boldsymbol{R}}$ values less than 1.10 were considered to be indicative of parameter convergence.

| **Explanatory Variable** | **Responding Variable** | **Standardized Slope Coefficient (ρ)** | **90% HPDI** | ***f*** | $\hat{\boldsymbol{R}}$ |
| --- | --- | --- | --- | --- | --- |
| SST | Menhaden | -0.16 | -0.56 – 0.23 | 0.75 | 1.02 |
| SST | Red drum | 0.05 | -0.24 – 0.35 | 0.61 | 1.00 |
| SST | Herring | -0.19 | -0.69 – 0.29 | 0.75 | 1.01 |
| SST | Caribbean | -0.14 | -0.25 – -0.03 | 0.98 | 1.00 |
| SST | Long-Line | 0.17 | -0.22 – 0.54 | 0.77 | 1.01 |
| SST | Trawl | 0.00 | -0.22 – 0.24 | 0.50 | 1.00 |
| Trawling | Menhaden | -0.14 | -0.25 – -0.03 | 0.98 | 1.00 |
| Trawling | Red drum | -0.13 | -0.38 – 0.12 | 0.80 | 1.01 |
| Trawling | Herring | -0.29 | -0.72 – 0.15 | 0.86 | 1.01 |
| Long-line | Menhaden | 0.36 | 0.12 – 0.63 | 0.99 | 1.01 |
| Long-line | Red drum | 0.00 | -0.17 – 0.17 | 0.51 | 1.02 |
| Long-line | Herring | -0.26 | -0.60 – 0.05 | 0.90 | 1.01 |

**Supplemental Table 4. (A)** Estimated standardized partial correlation coefficients (ρ) their associated error (90% Highest Posterior Density Intervals), and the proportion of the posterior distribution on the same side of zero as the mean (*f*) describing the associations between fluctuations in fish production (Atlantic menhaden, red drum, and herring 1^st^ year abundance), or fishery harvest (total landings in Caribbean, or in North Atlantic via trawling or long-line fishing) or pressure (number of hooks deployed in North Atlantic/Gulf of Mexico) throughout the Caribbean or North Atlantic on recovery probabilities for Royal terns banded as chicks in North Carolina or Virginia from 1960–2021. Parameter estimates with 90% of the posterior distributions on the same side of zero as the mean are presented in black text. (B) Estimated slope coefficients (β), their associated error (90% Highest Posterior Density Intervals), and, if applicable, the proportion of the posterior distribution on the same side of zero as the mean (*f*) describing the observed differences in breeding season detection, winter resighting, and recoveries between Royal terns banded in North Carolina and Virginia, and banded with an additional ancillary band or large plastic field readable (PFR) band. $\hat{\boldsymbol{R}}$ values less than 1.10 were considered to be indicative of parameter convergence.

| **(a) Explanatory Variable** | **Responding Variable** | **Standardized (ρ) Slope Coefficient** | **90% HPDI** | ***f*** | $\hat{\boldsymbol{R}}$ |
| --- | --- | --- | --- | --- | --- |
| Caribbean | Recovery Probability | -0.04 | -0.15 – 0.05 | 0.76 | 1.00 |
| Long-Line | Recovery Probability | 0.03 | -0.03 – 0.09 | 0.77 | 1.01 |
| Trawling | Recovery Probability | 0.07 | 0.01 – 0.13 | 0.97 | 1.01 |
| # Hooks | Recovery Probability | -0.02 | -0.09 – 0.04 | 0.74 | 1.04 |
| Menhaden | Recovery Probability | -0.01 | -0.05 – 0.04 | 0.57 | 1.00 |
| Red drum | Recovery Probability | -0.08 | -0.14 – -0.02 | 0.99 | 1.00 |
| Herring | Recovery Probability | 0.01 | -0.04 – 0.06 | 0.65 | 1.01 |
|  |  |  |  |  |  |
| **(b) Parameter** | **Responding Variable** | **(β) Slope Coefficient** | **90% HPDI** | ***f*** | $\hat{\boldsymbol{R}}$ |
| Intercept | Recovery Probability | -4.31 | -4.45 – -4.18 | NA | 1.01 |
| NC vs VA | Recovery Probability | 0.08 | 0.03 – 0.13 | 1.00 | 1.00 |
| Intercept | Resight Probability | -6.84 | -7.19 – -6.48 | NA | 1.00 |
| NC vs VA | Resight Probability | 0.69 | 0.61 – 0.77 | 1.00 | 1.00 |
| NC [Intercept] | Detection Probability | -7.30 | -7.84 – -6.73 | NA | 1.00 |
| VA [Intercept] | Detection Probability | -9.04 | -10.44 – -7.62 | NA | 1.00 |
| Ancillary | Recovery Probability | 0.29 | 0.14 – 0.44 | 1.00 | 1.00 |
| PFR | Recovery Probability | 0.36 | 0.16 – 0.56 | 1.00 | 1.00 |
| Ancillary | Resight Probability | 0.10 | -0.09 – 0.29 | 0.80 | 1.00 |
| PFR | Resight Probability | 2.24 | 2.04 – 2.44 | 1.00 | 1.00 |
| Ancillary | Detection Probability | 0.13 | -0.07 – 0.33 | 0.86 | 1.00 |
| PFR | Detection Probability | 8.47 | 6.48 – 10.43 | 1.00 | 1.00 |

**Supplemental Table 5.** Estimated standardized partial correlation coefficients (ρ), their associated error (90% Highest Posterior Density Intervals), and the proportion of the posterior distribution on the same side of zero as the mean (*f*) describing the associations between variation in climate (SST), fish production (Atlantic menhaden, red drum, and herring 1^st^ year abundance), or fishery harvest (total landings in Caribbean, or in North Atlantic via trawling or long-line fishing) or pressure (number of hooks deployed in North Atlantic/Gulf of Mexico) throughout the Caribbean or North Atlantic on age-specific (Pre-Breeding [Years 1 – 2], Sub-adult [Years 3 – 4], and Adult (5+ year old) mortality probabilities for Royal terns banded as chicks in North Carolina or Virginia from 1960–2021. Parameter estimates with 90% of the posterior distributions on the same side of zero as the mean are presented in black text. $\hat{\boldsymbol{R}}$ values less than 1.10 were considered to be indicative of parameter convergence.

| **Explanatory Variable** | **Responding Variable** | **Standardized Slope Coefficient (ρ)** | **90% HPDI** | ***f*** | $\hat{\boldsymbol{R}}$ |  |
| --- | --- | --- | --- | --- | --- | --- |
| SST | Pre-Breeding Mortality | 0.09 | 0.00 – 0.18 | 0.94 | 1.01 | |
| SST | Sub-adult Mortality | 0.02 | -0.09 – 0.12 | 0.59 | 1.03 | |
| SST | Adult Mortality | 0.01 | -0.11 – 0.12 | 0.54 | 1.00 | |
| Caribbean | Pre-Breeding Mortality | 0.02 | -0.13 – 0.17 | 0.58 | 1.01 | |
| Caribbean | Sub-adult Mortality | -0.01 | -0.19 – 0.16 | 0.53 | 1.06 | |
| Caribbean | Adult Mortality | -0.12 | -0.36 – 0.11 | 0.78 | 1.01 | |
| Long-Line | Pre-Breeding Mortality | -0.01 | -0.14 – 0.11 | 0.58 | 1.00 | |
| Long-Line | Sub-adult Mortality | -0.01 | -0.17 – 0.15 | 0.54 | 1.07 | |
| Long-Line | Adult Mortality | -0.13 | -0.39 – 0.09 | 0.81 | 1.01 | |
| Trawling | Pre-Breeding Mortality | -0.06 | -0.25 – 0.12 | 0.68 | 1.04 | |
| Trawling | Sub-adult Mortality | -0.01 | -0.17 – 0.18 | 0.53 | 1.08 | |
| Trawling | Adult Mortality | 0.12 | -0.09 – 0.34 | 0.81 | 1.01 | |
| # Hooks | Pre-Breeding Mortality | 0.02 | -0.04 – 0.09 | 0.73 | 1.01 | |
| # Hooks | Sub-adult Mortality | 0.13 | 0.03 – 0.25 | 0.97 | 1.07 | |
| # Hooks | Adult Mortality | 0.06 | -0.05 – 0.18 | 0.82 | 1.01 | |
| Menhaden | Pre-Breeding Mortality | -0.05 | -0.17 – 0.06 | 0.78 | 1.02 | |
| Menhaden | Sub-adult Mortality | -0.09 | -0.22 – 0.04 | 0.88 | 1.02 | |
| Menhaden | Adult Mortality | -0.12 | -0.27 – 0.03 | 0.91 | 1.01 | |
| Red drum | Pre-Breeding Mortality | 0.05 | -0.04 – 0.14 | 0.81 | 1.01 | |
| Red drum | Sub-adult Mortality | 0.01 | -0.09 – 0.12 | 0.54 | 1.06 | |
| Red drum | Adult Mortality | 0.03 | -0.11 – 0.16 | 0.62 | 1.00 | |
| Herring | Pre-Breeding Mortality | -0.04 | -0.20 – 0.12 | 0.65 | 1.02 | |
| Herring | Sub-adult Mortality | 0.04 | -0.10 – 0.18 | 0.69 | 1.01 | |
| Herring | Adult Mortality | 0.00 | -0.17 – 0.17 | 0.51 | 1.00 | |

**Supplemental Table 6.** Estimated parameter coefficients (β) and their associated error (90% Highest Posterior Density Intervals) for the mean estimates, intercepts or intercept-adjustment terms for the mortality, availability, and fidelity models. $\hat{\boldsymbol{R}}$ values less than 1.10 were considered to be indicative of parameter convergence.

| **Parameter** | **Parameter Coefficient (**β**)** | **90% HPDI** | $\hat{\boldsymbol{R}}$ | |
| --- | --- | --- | --- | --- |
| Pre-breeding mortality (linear-scale) | 0.15 | -0.06 – 0.32 | 1.03 |  |
| Second-year adjustment | -1.43 | -1.50 – -1.36 | 1.00 |  |
| Sub-Adult Mortality (linear-scale) | -1.79 | -2.09 – -1.55 | 1.04 |  |
| Fourth-year Adjustment | 0.03 | -0.10 – 0.17 | 1.00 |  |
| Adult Mortality (linear-scale) | -1.56 | -1.94 – -1.29 | 1.01 |  |
| Second Year Availability | 0.01 | 0.01 – 0.02 | 1.00 |  |
| Third Year Availability | 0.08 | 0.07 – 0.10 | 1.00 |  |
| Fourth Year Availability | 0.35 | 0.30 – 0.39 | 1.00 |  |
| Fifth Year Availability | 0.70 | 0.62 – 0.78 | 1.00 |  |
| Sixth Year Availability | 0.97 | 0.93 – 1.00 | 1.00 |  |
| State Fidelity | 0.98 | 0.98 – 0.99 | 1.00 |  |
| Mid-Atlantic Fidelity | 0.998 | 0.996 – 1.00 | 1.00 |  |

**Figure S1.** Detections of individuals on (**A**) the breeding grounds (yellow: North Carolina; blue: Virginia), or (**B**) during the non-breeding season in Florida; and (**C**) recoveries of individuals throughout the species geographic range were often highly variable from 1960–2021 potentially related to shifts in research efforts, access to photography equipment, and increased global knowledge of band reporting platforms.

**
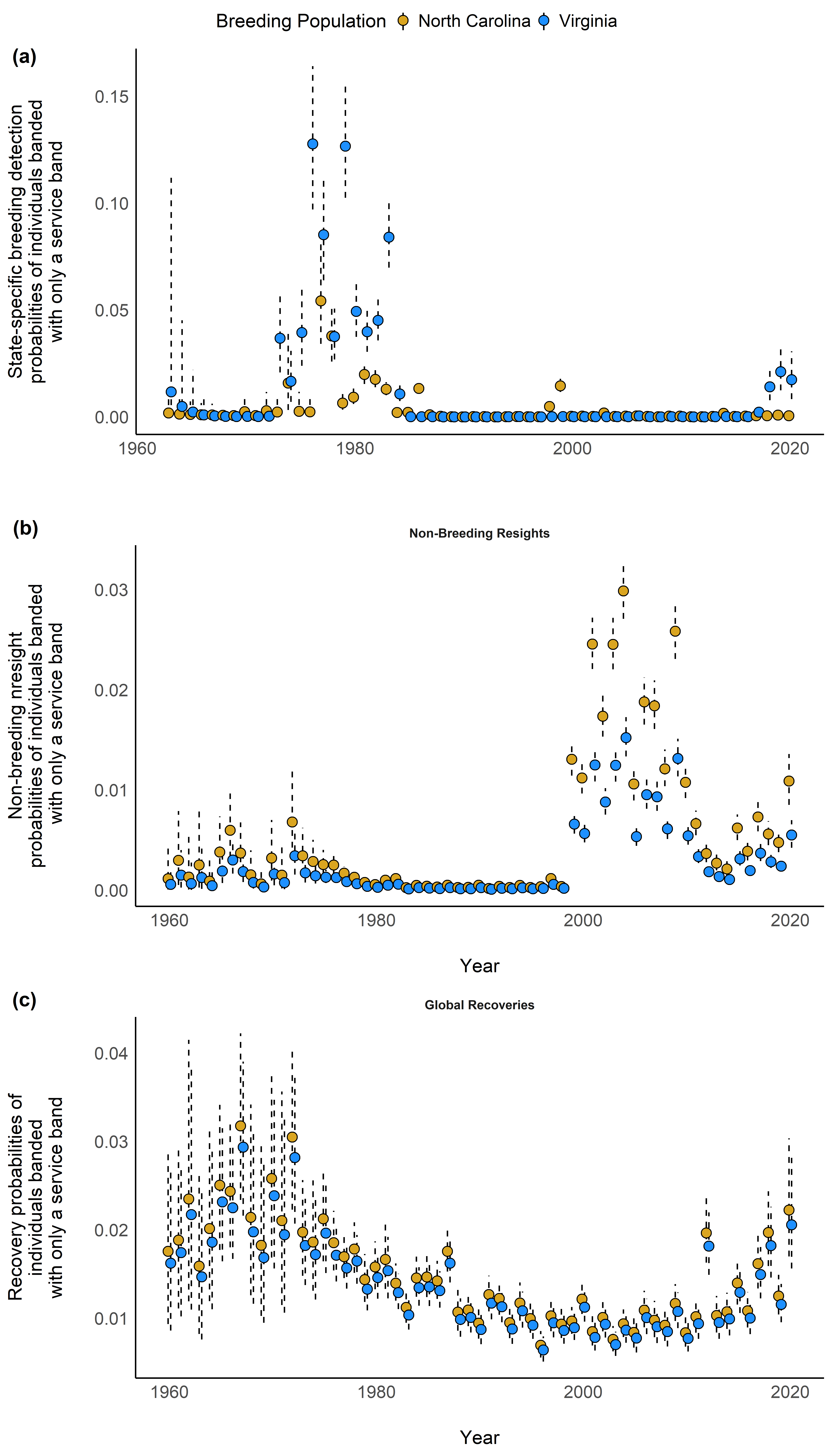
**

**Figure S2.**


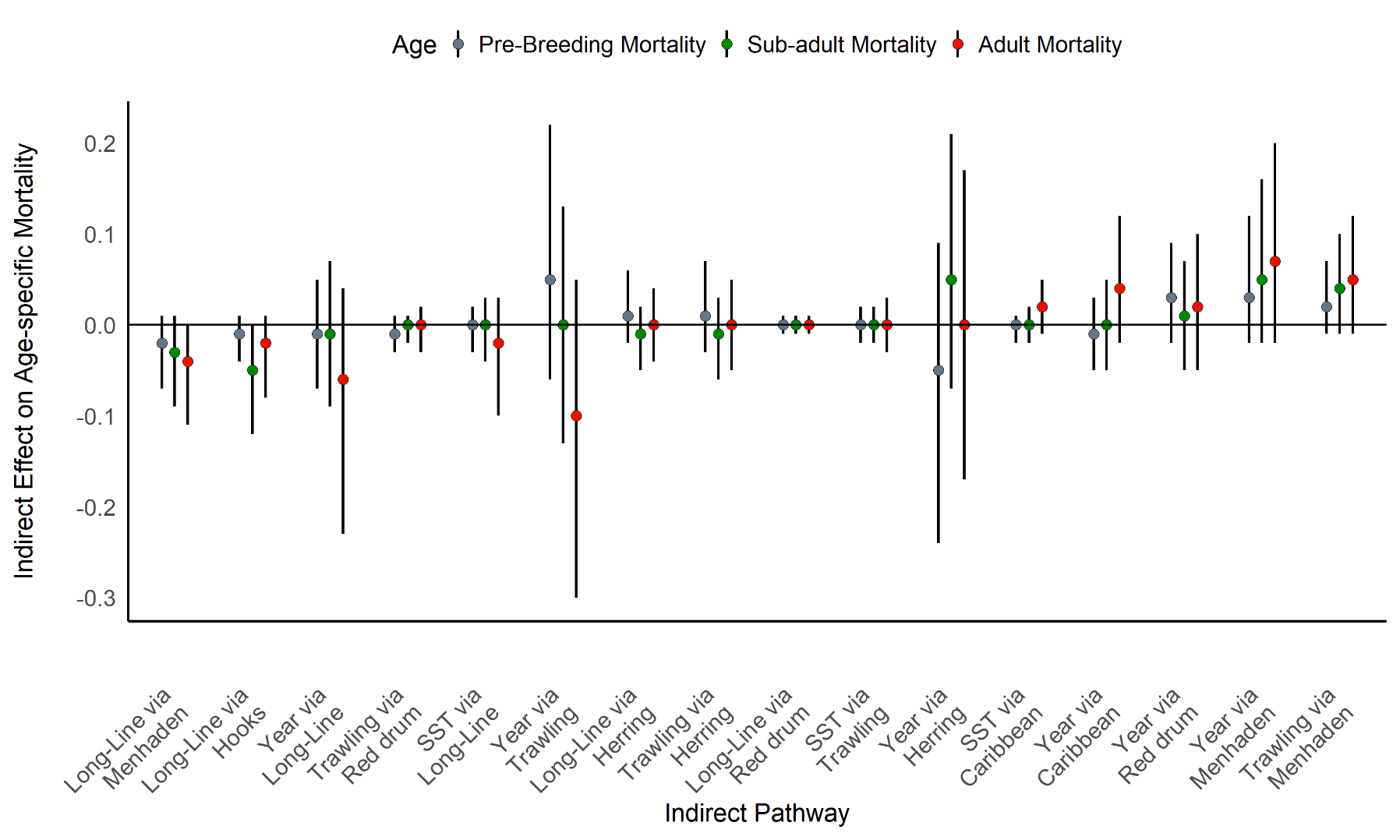

Supplement: Supplementary file 1 — Appendix S1 [file GCB-29-324-s002.docx]
