# Supplementary figures and images for: Climate change and commercial fishing practices codetermine survival of a long‐lived seabird
Source: Glob Chang Biol. 2022 Oct 22;29(2):324–40. doi: 10.1111/gcb.16482 (PMC10092490; doi:10.1111/gcb.16482)

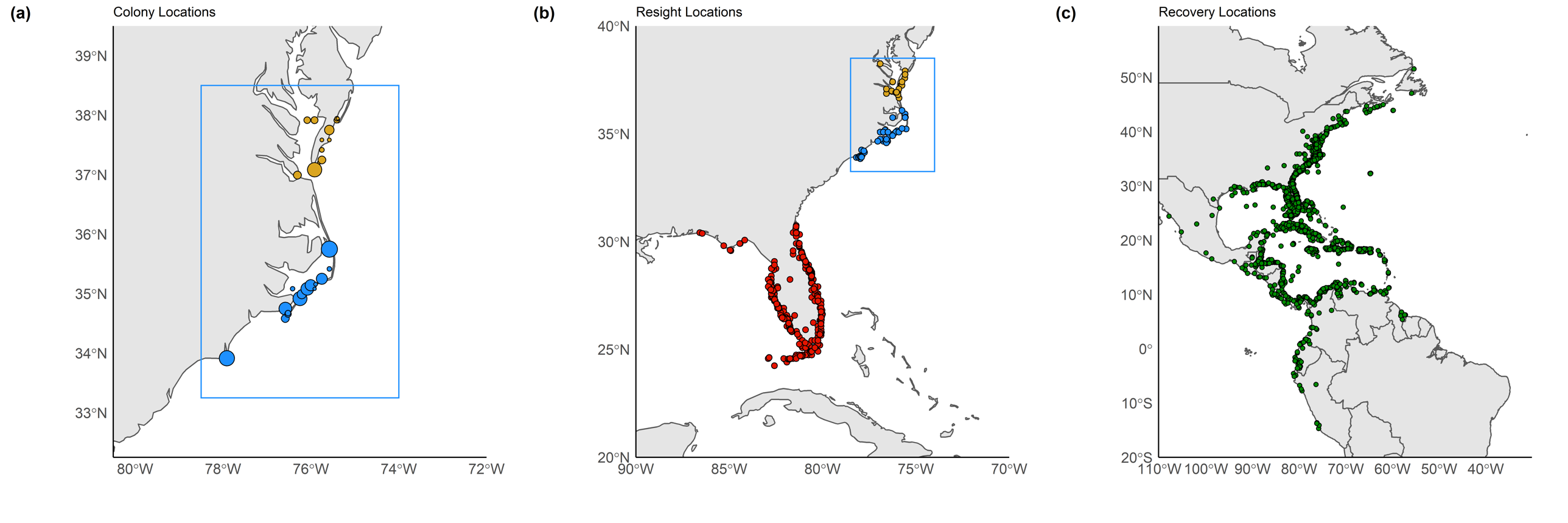

Supplement: Supplementary file 2 — Figure S2. The estimated indirect effects of environmental variables, and the directional pathway by which the variable was associated with pre‐breeding (gray), subadult (green), and adult (red) Royal tern mortality. Confidence intervals depict 90% HPDI. [file GCB-29-324-s001.png]
